# α-Synucleinopathy associated c-Abl activation causes p53-dependent autophagy impairment

**DOI:** 10.1186/s13024-020-00364-w

**Published:** 2020-04-16

**Authors:** Md. Razaul Karim, Elly E. Liao, Jaekwang Kim, Joyce Meints, Hector Martell Martinez, Olga Pletnikova, Juan C. Troncoso, Michael K. Lee

**Affiliations:** 1 Department of Neuroscience, University of Minnesota, Minneapolis, MN 55414 USA; 2 Present Address: Department of Neural Development and Disease, Korea Brain Research Institute (KBRI), Daegu, 41068 South Korea; 3 Department of Pathology, Johns Hopkins University School of Medicine, Baltimore, MD 21287 USA; 4 Institute for Translational Neuroscience, University of Minnesota, 2101 6th Street SE, Minneapolis, MN 55414 USA

**Keywords:** Neurodegeneration, Parkinson’s disease (PD), α-Synuclein, C-Abl, p53, Autophagy, mTOR, AMPK, Nilotinib, Pifithrin-α

## Abstract

**Background:**

Studies link c-Abl activation with the accumulation of pathogenic α-synuclein (αS) and neurodegeneration in Parkinson’s disease (PD). Currently, c-Abl, a tyrosine kinase activated by cellular stress, is thought to promote αS pathology by either directly phosphorylating αS or by causing autophagy deficits.

**Methods:**

αS overexpressing transgenic (Tg) mice were used in this study. A53T Tg mice that express high levels of human mutant A53TαS under the control of prion protein promoter. Two different approaches were used in this study. Natural aging and seeding model of synucleinopathy. In seeding model, intracortical/intrastriatal (IC/IS) stereotaxic injection of toxic lysates was done using tissue lysates from end-stage symptomatic mice. In this study, nilotinib and pifithrin-α was used as a c-Abl and p53 inhibitor, respectively. Both Tg and non-transgenic (nTg) mice from each group were subjected to nilotinib (10 mg/kg) or vehicle (DMSO) treatment. Frozen brain tissues from PD and control human cases were analyzed. In vitro cells study was implied for c-Abl/p53 genetic manipulation to uncover signal transduction.

**Results:**

Herein, we show that the pathologic effects of c-Abl in PD also involve activation of p53, as c-Abl activation in a transgenic mouse model of α-synucleinopathy (TgA53T) and human PD cases are associated with the increased p53 activation. Significantly, active p53 in TgA53T neurons accumulates in the cytosol, which may lead to inhibition of autophagy. Thus, we hypothesized that c-Abl-dependent p53 activation contributes to autophagy impairment in α-synucleinopathy. In support of the hypothesis, we show that c-Abl activation is sufficient to inhibit autophagy in p53-dependent manner. Moreover, inhibition of either c-Abl, using nilotinib, or p53, using pifithrin-α, was sufficient to increase autophagic flux in neuronal cells by inducing phosphorylation of AMP-activated kinase (AMPK), ULK1 activation, and down-regulation of mTORC1 signaling. Finally, we show that pharmacological attenuation of c-Abl activity by nilotinib treatment in the TgA53T mouse model reduces activation of p53, stimulates autophagy, decreases accumulation αS pathology, and delays disease onset.

**Conclusion:**

Collectively, our data show that c-Abl activation by α-synucleinopathy causes p53 dependent autophagy deficits and both c-Abl and p53 represent therapeutic target for PD.

## Background

Parkinson’s disease (PD) is a common late onset progressive neurodegenerative disease most characterized by movement disorder resulting from the loss of dopaminergic (DAergic) neurons in the substantia nigra pars compacta (SNpc) [1, 2]. In addition, PD is also characterized by the presence of protein inclusions known as Lewy bodies (LB) and Lewy neurites (LN), which are composed of aggregated α-synuclein (αS), in multiple neuronal populations [3–5]. While the etiology of PD is unknown in most cases, αS abnormalities are mechanistically linked to PD pathogenesis as mutations in αS cause PD in a small number of familial PD pedigrees [6, 7]. Currently, how αS abnormalities cause neuronal dysfunction and degeneration is not fully understood. However, studies have implicated oxidative stress in the pathogenesis of PD [8–10] and dysfunction in proteostasis [11, 12]. While oxidative stress in neurons has complex and multifaceted effects, recent reports suggest that activation of c-Abl, a non-receptor tyrosine kinase, can be stimulated by oxidative stress. And thus, may be linked to the pathogenesis of PD, Alzheimer’s disease (AD) and other neurodegenerative diseases [13, 14].

c-Abl is a tyrosine kinase known to be activated by cellular stressors, such as oxidative stress and DNA damage [15, 16]. c-Abl also functions to regulate many fundamental cellular processes, such as cell survival, migration, and growth factor signaling [17, 18]. While c-Abl abnormalities are linked to leukemia and other cancers, emerging studies implicate aberrant c-Abl activity in neurodegenerative disease [13]. In PD, c-Abl is activated in regions showing DAergic neurodegeneration, such as the striatum and SNpc, and inactivates parkin by phosphorylation [19–22]. Significantly, c-Abl activation is linked to αS pathology as increased αS expression in cells and Tg mice was associated with c-Abl activation, and inhibition of c-Abl [23] or the loss of c-Abl expression [24] leads to attenuation of αS levels and/or aggregation. Some of these studies implicate c-Abl as an inhibitor of autophagy [25, 26]. However, it is unknown how c-Abl regulates autophagy. Alternatively, a recent study proposed that direct phosphorylation of αS by c-Abl might be involved in the regulation of α-synucleinopathy by c-Abl [24, 27]. However, given that c-Abl-dependent tyrosine phosphorylated αS does form fibrils efficiently in vitro [28, 29], it is unclear that increased c-Abl activation can directly increase αS aggregation. Finally, it is currently unknown if pharmacological inhibition of c-Abl in adult animals can attenuate onset and progression of overt α-synucleinopathy.

In previous studies linking α-synucleinopathy and c-Abl activation, a key pathway traditionally associated with the c-Abl activation was not addressed. Specifically, c-Abl can regulate p53 activity via phosphorylation of Mdm2/Hdm2 [30], an E3 ligase responsible for degradation of p53. In the past, we showed that in the TgA53T model, neurodegeneration is associated with an increase in neuronal p53 expression [31]. Thus, we examined whether the αS-dependent c-Abl activation was associated with increased p53 activation. Herein, we show increased activation of both c-Abl and p53 with α-synucleinopathy in a TgA53T mouse model of α-synucleinopathy [32] and in human PD cases. Fundamentally, a significant fraction of active p53 was mislocalized to the cytosolic compartment of neurons exhibiting αS pathology, a phenomenon shown to inhibit autophagy [33]. Indeed, we show that c-Abl-dependent inhibition of autophagy is p53 dependent. Further, both c-Abl and p53 activity is positively associated with mTOR activity and inversely associated with AMPK/ULK1 activity, showing that c-Abl and p53 directly impact the pathways relevant to autophagy regulation. Finally, we show that c-Abl-dependent pathway is a significant target for therapeutic intervention as pharmacological inhibition of c-Abl delays disease onset in two independent Tg mouse models of α-synucleinopathy. Our data identify a novel pathway for regulation of autophagy in α-synucleinopathy and support the development of c-Abl and p53 inhibitors for disease modifying therapies for PD and other α-synucleinopathies.

## Methods

### Animals

The generation of transgenic (Tg) mice (TgA53T) that express high levels of human mutant A53TαS under the control of a mouse prion protein promoter have been described previously [31, 32, 34, 35]. Mice expressing the A53TαS develop progressive neurological dysfunction ~ 12 months of age, which rapidly progress to end stage paralysis within 14–21 days following initial onset of symptoms. For this study Tg mice were evaluated at early stage defined by slight instability, bradykinesia, and ataxia. End-stage was defined by the onset of paralysis in all limbs. Asymptomatic mice were 10–14 months old but did not display any motor deficits. Age matched non-transgenic (nTg) littermates were used as controls. At 9 months of age, a cohort of animals including both Tg and nTg were subjected to treatment with 10 mg/kg Nilotinib or DMSO (Vehicle) control. Animals were dosed by intraperitoneal (i.p.) injections three times weekly until animals displayed end-stage phenotypes described above. All experimental protocols involving mice were in strict adherence to the NIH Animal Care and Guidelines and were approved by the Institutional Animal Care and Use Committee at the University of Minnesota. We used about equal number of male and female mice in this study.

### Induction of α-synucleinopathy by inoculation

Lysates used for all injections were generated from tissues of *TgA53T* mice at either 4 months of age (asymptomatic) or end-stage. Brainstem (BST) and spinal cord (SPC) were combined and processed together, as both regions demonstrate robust αS pathology at the end-stage [32]. The 3000×g lysate were prepared as previously described [36].

All stereotaxic injections were performed unilaterally into the right hemisphere in *HuA53TaS* mice at aged 6 months. Animals were anesthetized by Ketamine/Xylazine mixture (100/10 mg/kg, i.p.) and stereotaxically injected with 2.5 μg of total protein in 2.5 μl. The injections occurred at a rate of 0.1 μl per minute using a 28 g needle attached via tubing to a Hamilton syringe controlled by a constant pressure syringe pump (Harvard Apparatus, Holliston, MA). The stereotaxic coordinates used for the injection sites were as follows: intracortical/intrastriatal (IC/IS): 2.0 mm lateral from the midline, + 0.2 mm relative to bregma, and 0.8 and 2.6 mm deep from the dura. Two weeks following the inoculation surgery, the animals were subjected to i.p. injections of either Nilotinib (10 mg/kg) or Vehicle (DMSO) three times weekly as described above. Animals were euthanized at either pre-determined time points or upon complete hind-limb paralysis by overdose of isoflurane inhalation and tissues were harvested for either biochemistry or immunohistochemistry.

### Tissues from human PD and control cases

Fresh frozen brain tissues (Pons) of PD and control human cases were obtained from the Brain Resource Center (Department of Pathology, Johns Hopkins University School of Medicine). The pathological characterizations of the tissues were done as described [37, 38].

### Antibodies

The list of primary antibodies used in this study are listed in the supplementary table (Additional file 1: Table S1).

### Immunoblot analysis of protein expression

Relative protein levels of αS, c-Abl, p53, Mdm2 and other proteins were determined from whole tissue extracts by quantitative immunoblots analysis [32, 37] using chemiluminescence detection of horseradish peroxidase-conjugated secondary antibodies. Images were captured using the GE Imagequant LAS-4000 (GE Healthcare, Waukesha, WI) and the Imagequant software (GE) was used to determine the intensity of the immunoreactive bands.

### Immunoprecipitations

Immunoprecipitations were conducted using the Crosslink Immunoprecipitation Kit (Thermo Scientific, Rockford, IL) as previously described [37, 39]. Briefly, 1 μg of Mdm2 antibody was cross-linked using 2 mM DSS to protein G agarose. Bound proteins were freed from the beads by SDS-sample buffer before separation by SDS-PAGE.

### Immunohistochemistry

Brains and spinal cords from appropriate mice littermates were fixed in 4% paraformaldehyde (PFA) and embedded in paraffin. Sections were cut at 7 μm and immunostained using an immunoperoxidase method with diaminobenzidine (DAB) as previously described [31, 40, 41] or double immunostaining followed by confocal imaging. Alex fluor (AF-647 or AF-488) conjugated secondary antibody was used. Alternatively, fixed brains were immersed in 30% sucrose solution and prepared for frozen sliding microtome cut at 40 μm followed by immunostaining of free-floating sections [41].

### Cell culture

BE(2)-M17 cells expressing human αS under the regulation of doxycycline [39] (Colla et al., 2012), Hippocampal neuronal cell lines, and human embryonic kidney cell line (HEK-293) were used in this study. Cells were transfected with pcDNA3.1-c-Abl-PP or pcDNA3.1-c-Abl-KDM plasmid [42, 43] using Lipofectamine LTX. A double proline mutation in the regulatory ‘linker’ region between the SH2 and kinase domain (P242E/P249E) confers constitutive activity to c-Abl, and the kinase dead mutant (KDM) bearing the additional K290R mutation [44]. A p53 and scramble SiRNA (GS 7157, Qiagen) were done by using DharmaFECT1 transfection reagent (T-2001-02). Nilotinib and pifithrin-α were administered. Cells were washed with PBS and harvested in TNES buffer for protein analysis as described above [39, 40].

### Statistical analysis

To test for statistical significance between treatment groups, data was analyzed by one-way or two-way analysis of variance (ANOVA) followed by a Multiple Comparison Post-hoc test (Tukey’s/Dunnett’s/Bonferroni’s), or Student’s t test using GraphPad PRISM Software (7.0). All the data are expressed as means ± S.E. Probability (p) values less than 0.05 were considered significantly different.

## Results

### α-Synucleinopathy leads to c-Abl and p53 activation

Previous studies implicate c-Abl in the pathogenesis of Parkinson’s disease (PD). c-Abl can inactivate parkin [45] or directly phosphorylates α-synuclein (αS) [24, 27]. However, because c-Abl is known to increase p53 activation via inactivation of Mdm2/Hdm2, an E3 ubiquitin-ligase for p53 [30, 46], we propose that the pathogenic action of c-Abl in α-synucleinopathy may involve p53. Thus, we determined if both c-Abl and p53 are coordinately activated in the TgA53T model of α-synucleinopathy [32]. Progressive neurodegenerative diseases in these mice are associated with signs of oxidative stress, as indicated by mitochondrial abnormalities [31] and accumulation of oxidative damage, including accumulations of 3-nitrotyrosine [47] (Additional file 1: Figure S1), conditions that promote both c-Abl and p53 activation.

**Fig. 1:**
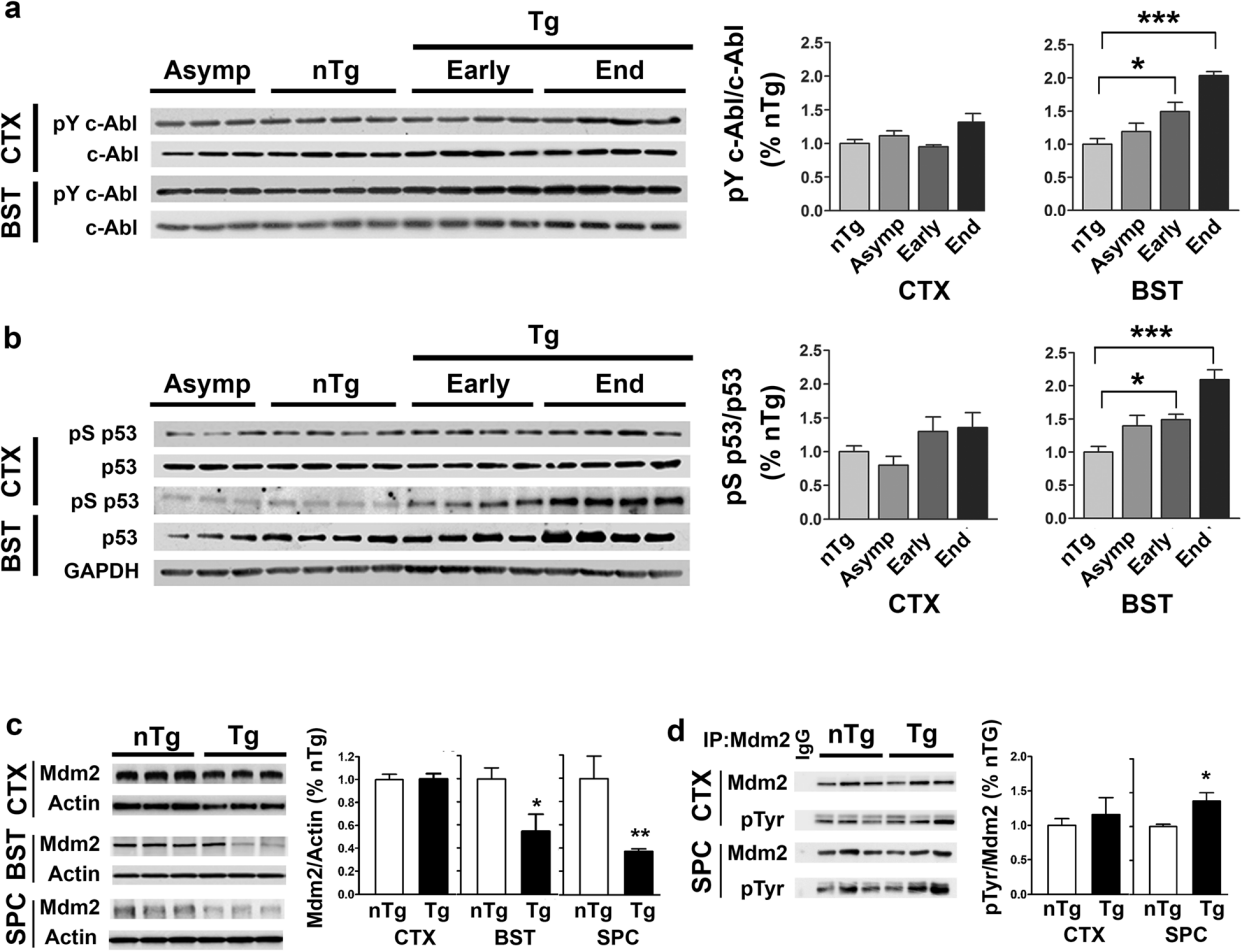
Activation of c-Abl, p53 and Mdm2 with α-synucleinopathy in TgA53T mice. **a, b** Levels of phospho-Tyr_245_ c-Abl (pY245c-Abl) (**a**) and phospho-Ser_15_P53 (pS15p53) (**b**) in the cortex (CTX) and brainstem (BST) of mice at various disease states: Non-transgenic (nTg), aged asymptomatic TgA53T mice (Asymp), and early stage/end stage (Early/End) symptomatic TgA53T mice. Quantitative analysis graphs for corresponding protein levels in BST and CTX are shown on the right side of each figure. Levels of phosphorylated c-Abl (**a**) or p53 (**b**) were not changed in the cortex, but significantly increased in BST region of symptomatic (Early and End) animals. All values are mean ± SEM, *n* = 3–4 animal per group. ***p < 0.001, **p < 0.01, *p < 0.05 compared with the nTg littermate mice, one-way ANOVA followed by Dunnett’s multiple comparison test. **c, d** Activation of Mdm2 in A53TαSyn Tg mice. **c** Levels of Mdm2 in cortex (CTX), brainstem (BST) and spinal cord (SPC) of non-transgenic (nTg), and end stage transgenic (Tg) mice. **d** Tyrosine phosphorylated Mdm2 was evaluated by Mdm2 immunoprecipitation followed by immunoblotting using an anti-phospho-Tyr antibody. Despite the reduced levels of Mdm2 in the SPC (**c**), Increased pTyr-Mdm2 is seen with SPC of TgA53T compared to the nTg mice. Bar graph for corresponding protein levels were shown next to each figure. All values are mean ± SEM, *n* = 3–4 animals. **p < 0.01 and *p < 0.05 compared with the nTg littermate by Student’s *t* test

Analysis of symptomatic TgA53T mice at early and end-stage of disease [39, 40] show that while the overall c-Abl levels are not different from age matched non-Tg (nTg) mice, there is a significant increase in the level of c-Abl phosphorylated at Tyr_245_, an indicator of active c-Abl, in regions affected by α-synucleinopathy [brainstem (Fig. 1) and spinal cord (Additional file 1 : Figure S2)]. We can exclude c-Abl activation as a consequence of αS overexpression based on the facts that: 1) c-Abl activation is absent in TgA53T cortex (Fig. 1a), which lack significant αS pathology despite high levels of mutant αS expression [32]; and 2) analysis of brain stem regions from asymptomatic TgA53T mice lacking αS pathology do not show increased c-Abl (Fig. 1a). Collectively, these data show that c-Abl activation in TgA53T mice require overt αS pathology.

Analysis of p53 levels show that the relative levels of active p53, the phospho-Ser_15_ (pS15p53), mirrors the increase in the c-Abl activation (Fig. 1b**,** Additional file 1: Figure S2) as the abundance of active p53 is higher in the subcortical areas of symptomatic TgA53T mice (Fig. 1b; Additional file 1: Figure S2) and progressively increases with the progression of disease (i.e., end-stage showing higher levels of active p53 than the early stage symptomatic TgA53T mice). As with the activation of c-Abl, activation of p53 is not due to simple overexpression of the mutant αS as the levels of active p53 in TgA53T mice was not increased in absence of significant α-synucleinopathy, such as in cortex and asymptomatic animals (Fig. 1b).

We then examined whether the increased activation of c-Abl and p53 was associated with abnormalities in Mdm2. Analysis of Mdm2 in end-stage TgA53T and nTg littermate mice show reduced total Mdm2 levels in both the brainstem and spinal cord regions but not in the cortex (Fig. 1c). We also examined whether the reduced Mdm2 levels in TgA53T was associated with increased tyrosine phosphorylation by immunoblot analysis of immunoprecipitated Mdm2 for phosphor-Tyr (Fig. 1d; Additional file 1: Figure S3). The results show that, consistent with Mdm2 being a substrate for c-Abl, the reduction in Mdm2 was associated with increased tyrosine phosphorylation of Mdm2 (Fig. 1d).

### Cellular localization of active c-Abl and p53 is coincident with neuronal α-synucleinopathy

**Fig. 2:**
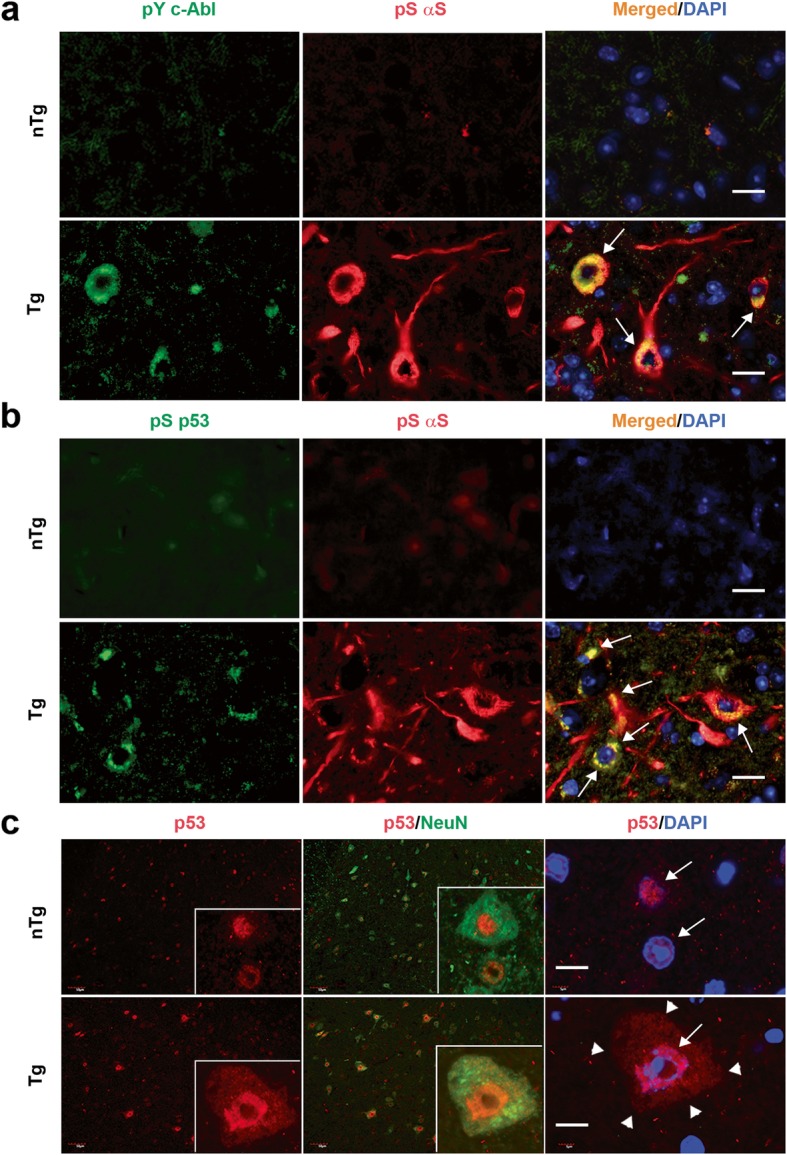
Activated c-Abl and p53 accumulates in neurons exhibiting αS pathology. **a, b** Pathological αS accumulation in the brainstem of end-stage TgA53T Hu αS mice were detected using anti-pS129αS antibody (Red). The sections were also double immune-stained for activated c-Abl (pY245c-Abl, Green) (**a**) or activated p53 (pS15p53, Green) (**b**). Arrows indicate colocalization of active c-Abl (**a**) and p53 (**b**) with neurons that accumulate pS129αS. No significant staining for pS129αS, pY245c-Abl, or pS15p53 are seen in nTg mice. Scale, 20 μm. **c.** Spinal cord (SPC) sections from nTg and end stage TgA53T mice were immunostained using a pan-p53 antibody followed by a confocal fluorescence imaging. Note the overall increase in the p53 immunoreactivity in TgA53T mice compared to nTg mice. Arrows shows the nuclear localization of p53 in both nTg and Tg. Arrowhead shows the cytosolic localization of p53 seen only in the TgA53T mice. Scale, 10 μm

To determine if c-Abl and p53 activation occurs in neurons exhibiting αS pathology, we performed double immunofluorescence analysis for αS pathology (pS129αS) and either pY245c-Abl or pS15p53 in brainstem from TgA53T and nTg mice. As expected, nTg mice did not show expression of pS129αS, pY245c-Abl, or pS15p53 (Fig. 2a,b). In contrast, TgA53T mice show clear neuronal expression of pS129αS, pY245c-Abl, and pS15p53 (Fig. 2a,b). Moreover, virtually all pY245c-Abl and pS15p53 expression was associated with neurons containing pS129αS (Fig. 2a,b). These results demonstrate that the increased c-Abl and p53 selectively occurs in neurons harboring αS pathology.

A notable aspect of the pS15p53 immunostaining in TgA53T neurons was that, instead of the usual nuclear localization of p53, pS15p53 was prominently localized to cytosolic compartment (Fig. 2b, arrow). Analysis of total p53 distribution using a pan-p53 antibody also showed an substantial increase in the neuronal p53 staining in TgA53T mice compared to nTg mice (Fig. 2c), with the increased nuclear p53 staining in TgA53T mice (Fig. 2c, arrow). Significantly, in addition to the increased nuclear p53 in TgA53T neurons, there was clear cytosolic accumulation of p53 in the TgA53T neurons (Fig. 2c, arrowhead), whereas p53 was restricted to nucleus (arrow) in nTg neurons. Analysis of p53 localization in other brain regions affected by αS pathology also show dramatic cytosolic mislocalization of p53, where p53 accumulation extends to the proximal neurites (Additional file 1: Figure S4). These results are consistent with our hypothesis that the active pS15p53 accumulates in the cytosol because Mdm2 does not target p53 for proteasomal degradation.

### α-Synucleinopathy in TgA53T mice is associated with dysregulation of autophagy-lysosomal process

**Fig. 3:**
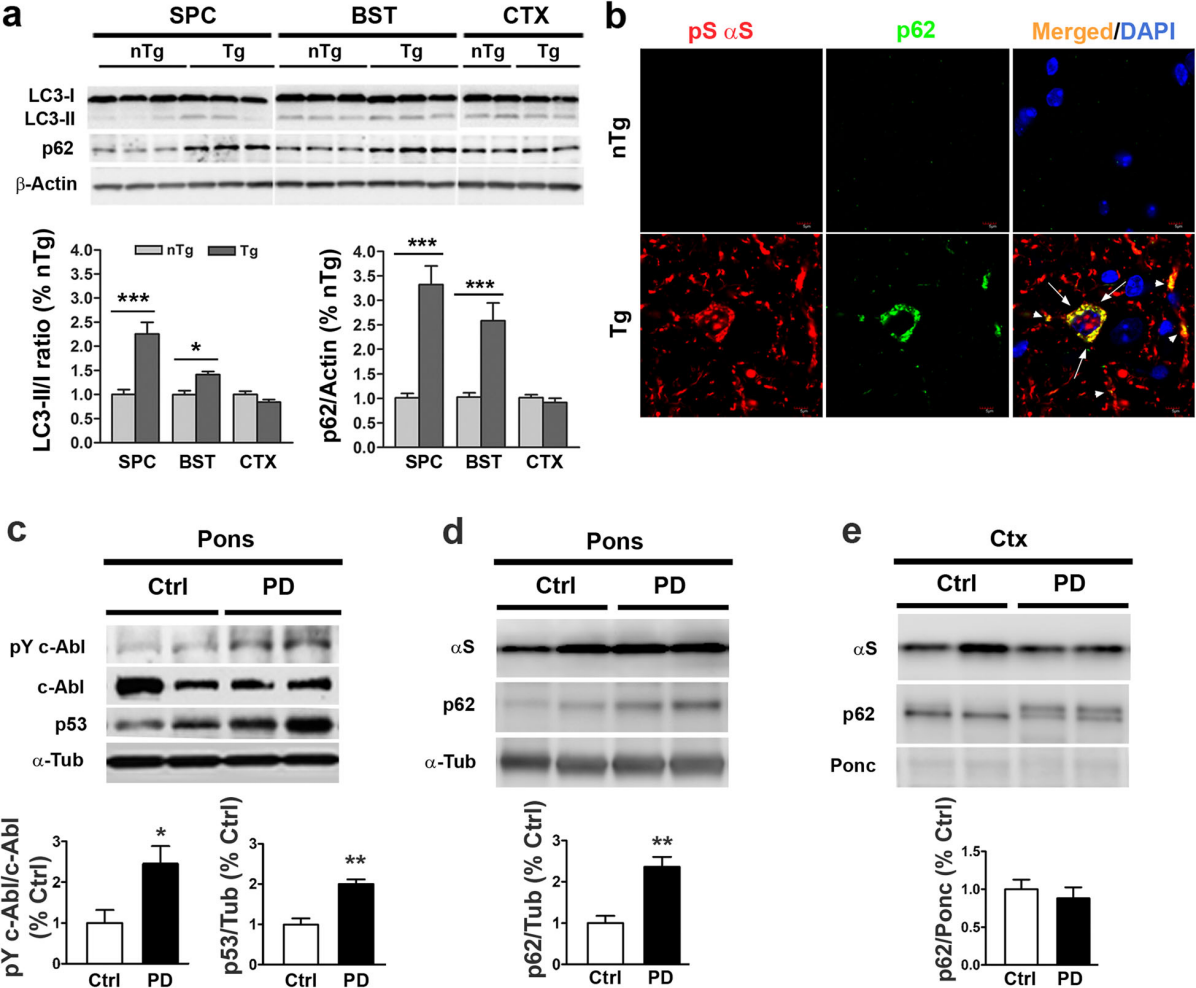
Aberrant accumulation of autophagy markers in TgA53T neurons with αS pathology. **a** Western blot analysis of LC3-I, LC3-II and p62 in symptomatic TgA53T mice and littermate nTg mice. The graphs (below) show the LC3-II/I ratio and p62 levels derived from the immunoblots. The brain regions analyzed are spinal cord (SPC), brainstem (BST), and cortex (CTX). There is clear increase in the levels of LC3-II and p62 in brain regions (SPC, BST) associated with the αS pathology, while the lack of pathology in CTX is associated with no changes in LC3-II and p62 levels. Values are mean ± SEM; *n* = 6–8. There was a significant effect (*p* < 0.001) for species (nTg-Tg), brain location (SPC-BST-CTX) and interaction for both LC3 ratio and p62. ***p < 0.001, *p < 0.05, Two-way ANOVA followed by Bonferroni multiple posttest. **b** Paraffin sections of brainstem (BST) were double immunofluorescence stained for p62 (green) and pS129αS (red). Arrows show that accumulated p62 is selective localized to the neurons that accumulate pS129αS. Arrowheads show similar p62 accumulation in neurites. Significant accumulation of p62 suggest that autophagosome clearance might be impaired in TgA53T neurons. Scale bar, 10 μm. **c, d, e** c-Abl activation and p62 accumulation in human PD cases. Levels of phospho-Tyr412 c-Abl (pY412c-Abl), c-Abl and p53 (**c**) and αS, p62 (**d**) in pons (**c, d**), and αS, p62 in cortex (CTX, **e**) of human postmortem tissue lysates from control (Ctrl) and PD cases were evaluated by Western immunoblot analysis. Bar shows the relative abundance of each protein (Mean ± SEM; *n* = 3–5). **p < 0.01, *p < 0.05, Student’s *t* test

The cytosolic accumulation of active p53 with α-synucleinopathy is of interest because cytosolic p53, but not nuclear p53, is implicated as a negative regulator of autophagy [33]. Thus, we asked whether robust cytosolic neuronal accumulation of p53 in TgA53T mice is associated with signs of impaired autophagy. As an indicator of autophagic status in TgA53T mice, we examined the expression of p62, an adaptor molecule and recruit substrates to autophagosomes, and LC3-II, a marker of autophagosome biogenesis and interact with p62 [48]. Our results show that brain regions with αS pathology in TgA53T mice exhibit significant increases in both p62 and LC3-II levels compared to the levels in nTg mice (Fig. 3a). We also examined whether abnormal autophagy, as indicated by p62 accumulation is coincident with the presence of αS pathology. Double immunofluorescence analysis shows that the increase p62 occurs in neurons containing αS pathology (Fig. 3b**;** Additional file 1: Figure S5). Normally, p62 binds with LC3-II to transport defective cargo to the autophagosome for degradation [49]. Thus, when autophagy is inhibited, particularly at the level of autophagosome fusion with the lysosomes, both p62 and LC3-II accumulates [50]. The fact that both LC3-II and p62 accumulates indicate that α-synucleinopathy in TgA53T mice is associated with the dysfunctional autophagy, particularly at the step of autophagosome clearance.

### Human PD cases exhibit c-Abl activation and p62 accumulation

To determine whether increased c-Abl and p53 activation also occurs in human α-synucleinopathy, we examined human postmortem brain tissue from PD cases (Fig. 3c). To correlate c-Abl/p53 activation with αS pathology, independent of ongoing dopaminergic neurodegeneration, we examined the Pontine region from human subjects as this region shows most significant αS pathology in PD cases [38, 51]. As in TgA53T mice, the presence of αS pathology is associated with significant increases in the levels of active c-Abl and total p53 in PD cases compared to age matched controls (Fig. 3c). Previously reported, increased p53 occurs in the cytosolic compartments of neurons in variety of neurodegenerative diseases, including PD, AD, DLBD and ALS [52]. Collectively, our results and previous p53 localization study [52] support the view that TgA53T model is reflecting the abnormalities seen in human α-synucleinopathy. Consistent with this view, analysis of the pontine region also reveals a significant increase in the level of autophagy substrate p62 in the PD cases compared to the age matched controls (Fig. 3d). In contrast, p62 accumulation is absent in cortex of PD cases (Fig. 3e), suggesting autophagy impairment in human PD cases is also associated with αS pathology.

### C-Abl activation causes p53-dependent autophagy defect

**Fig. 4:**
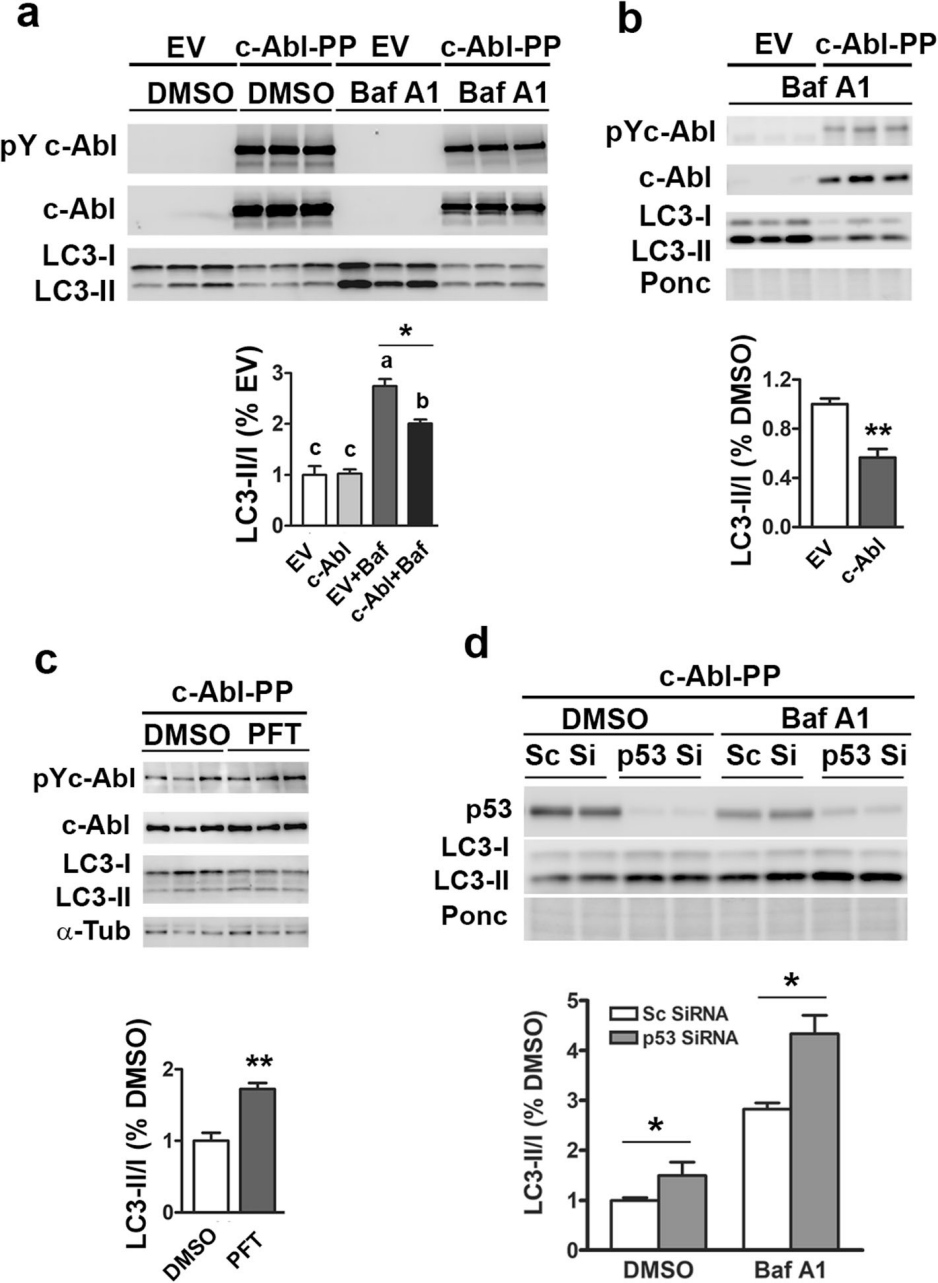
Constitutively active c-Abl causes p53-dependent autophagy deficit. **a, b, c** Human embryonic kidney cells, HEK-293 (**a, c**) or M17 human neuroblastoma cells (**b**) were transfected with empty vector (EV) or constitutively active c-Abl (c-Abl-PP) and, at 24 h post-transfection, the cells were treated with DMSO or Bafilomycin A1. Total lysates were immunoblotted for pY412c-Abl, c-Abl, and LC3. The quantitative analyses of the immunoblots show means ± SEM (n = 3). The results show that the expression of c-Abl-PP leads to suppression of autophagy (**a, b**). **c, d** HEK293 cells expressing c-Abl-PP were treated with a p53-inhibitor (pifithrin-α; PFT) (**c**) or p53 siRNA (Si) (**d**). In (**d**) scrambled siRNA (Sc Si) were used as a control and cells were further treated with DMSO or Bafilomycin A1. Analysis of pY412c-Abl, c-Abl, p53 and LC3 show that both pharmacological (PFT, **c**) or genetic (p53 Si, d) inhibition of p53 reverses the effects of c-Abl-PP. **a**^a,b^Significantly different from c, *p* < 0.01, *p < 0.05, One-way ANOVA with Tukey’s multiple comparison posttest. **b, c, d** *p < 0.05; **p < 0.01, Student’s *t* test

Thus far, our results support the hypothesis that c-Abl-dependent accumulation of p53 leads to autophagy defect in TgA53T mouse model. To more directly test the link between c-Abl, p53, and autophagy, we used cellular models to test if increased c-Abl activity is sufficient to inhibit autophagy in a p53-dependent manner. First, we used constitutively active c-Abl (c-Abl-PP) [42, 43] to drive c-Abl activity in HEK-293 cells and M17 neuroblastoma cells (Fig. 4**;** Additional file 1: Figure S6, S7, S8). In both cell types, expression of c-Abl-PP lead to significant increase in c-Abl activity, as indicated by the increased Tyr_412_ autophosphorylation of c-Abl (Figs. 4**;** Additional file 1: Figure S6, S7, S8). Increased c-Abl activity was also associated with clear deficits in the autophagy flux compared to the empty vector alone in HEK293 cells (Fig. 4a**;** Additional file 1: Figure S6) and in M17 mouse neuroblastoma cells (Fig. 4b). In addition to basal autophagy, activation of c-Abl significantly inhibited autophagy following nutrient starvation (Additional file 1: Figure S7a), demonstrating that c-Abl regulates the key steps in autophagy involved in multiple types autophagy activation. Inhibition of autophagy by c-Abl requires the kinase activity as autophagy was not inhibited by the kinase-dead c-Abl (KDM) [43] (Additional file 1: Figure S7b). Overall, these results demonstrate that increased c-Abl activation is sufficient to inhibit multiple types of autophagy. Because c-Abl-PP overexpression lead to defects in autophagy flux and reductions in both LC3-I and LC3-II levels, c-Abl may inhibit early stages of autophagosome formation by reducing LC3 expression.

We then examined the role of p53 in the c-Abl dependent inhibition of autophagy. First, expression of constitutively active c-Abl-PP expression leads to significant decrease in Mdm-2 levels and increased p53 levels (Additional file 1: Figure S8). More important, active p53 is required for c-Abl dependent inhibition of autophagy, as inhibition of p53 by pifithrin-α (PFT) was able to block autophagy defect in c-Abl-PP expressing cells (Fig. 4c). To further demonstrate that p53 mediates autophagy inhibition by c-Abl, we used siRNA to knockdown p53 (Fig. 4d). Compared to the scrambled siRNA, p53 siRNA was able achieve > 80% reduction in p53 levels. Analysis of LC3-I and LC3-II show that as with PFT treated cells, p53 knockdown was able to rescue defects in autophagy flux in c-Abl-PP transfected cells (Fig. 4D).

**Fig. 5:**
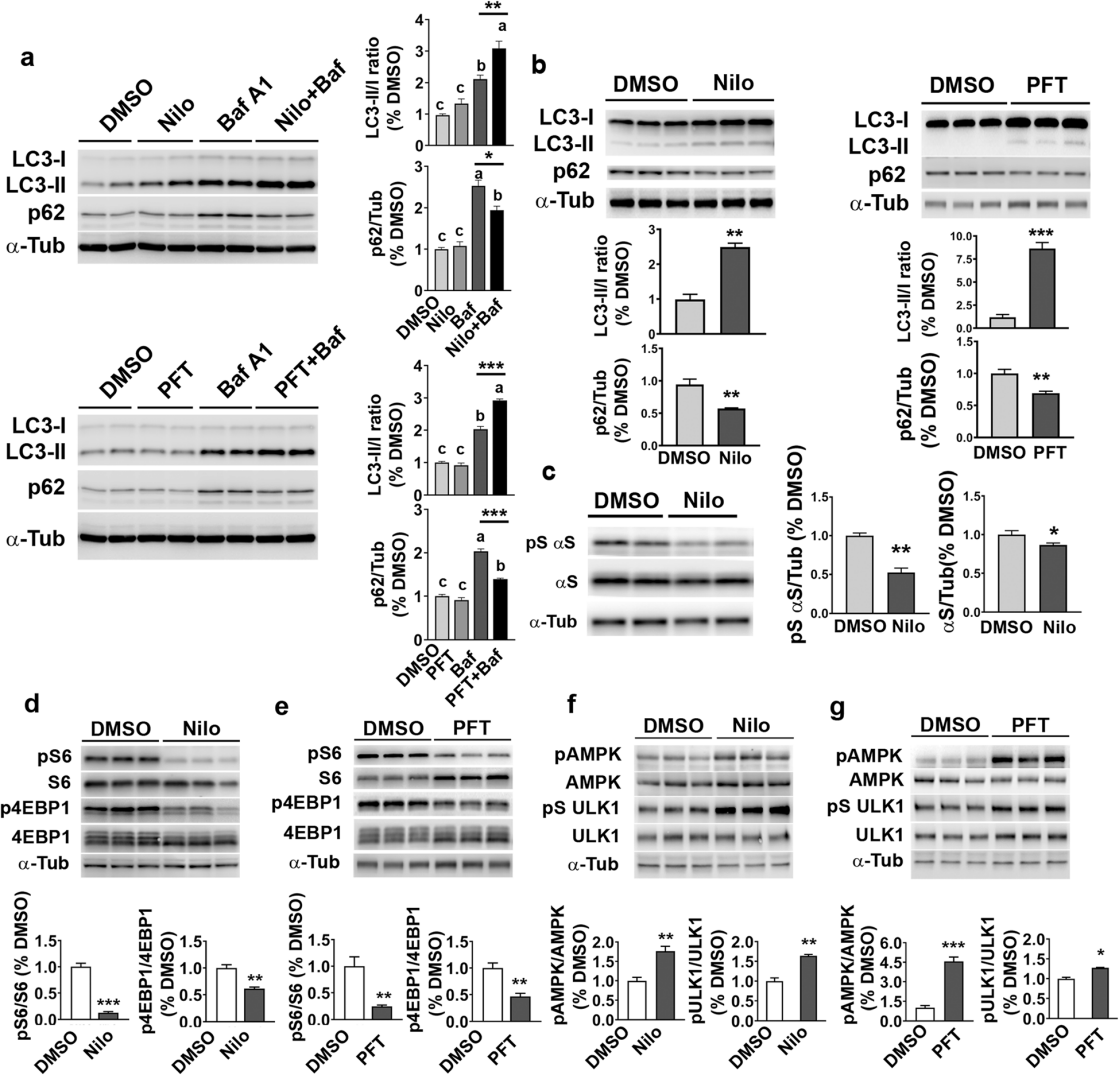
Inhibition of c-Abl and p53 modulate basal autophagy, αS levels and regulate AMPK/mTOR signaling in neuronal cells. Hippocampal CLU neuronal cell lines (**a**) and M17 (A53T) human neuroblastoma cells (**b, c**) were treated with 2.5 μM nilotinib (Nilo) or 30 μM pifithrin-α (PFT) for 6 h (M17 cells) and 12 h (CLU cells). To determine autophagic flux, some cells were treated with 100 nM bafilomycin A1(BafA1) for the last 5 h. Following the treatments, the cell lysates were analyzed for LC3 and p62 (**a, b**) or pS129αS/total αS (**c**). α-Tub (α-tubulin) was used as a loading control. Data are means ±SEM from n = 3 separate experiments. **a** Analysis of BafA1 treated cells show that Nilo treatment leads to greater LC3-II levels following BafA1 treatment, indicating that c-Abl inhibition enhances basal autophagy. ^a,b^ Significantly different from c, *p* < 0.001, **p < 0.01; ***p < 0.001, One-way ANOVA followed by Tukey’s test. n = 3 per treatment. **b, c** Total lysates from M17 cells expressing human αS transgene were immunoblotted for LC3 and p62 (**b**) or pS129αS/total αS (**c**) following Nilo or PFT treatment. Quantitative analysis shows that both Nilo and PFT treatment increases basal LC3-II levels (**b**)**.** Further, Nilo treatment reduces pS129αS levels (**c**). *p < 0.05; **p < 0.01; ***p < 0.001, Student’s *t* test. n = 3 per treatment. **d, e, f, g** Modulation of AMPK/mTOR signaling by c-Abl and p53 inhibition. Differentiated M17 (A53T) human neuroblastoma cells were treated with c-Abl inhibitor (2.5 μM Nilo, **d** and **f**) or p53 inhibitor (30 μM PFT, **e** and **g**) for 6 h. **d, e** Total lysates were immunoblotted for pS6, S6, p4EBP1, 4-EBP1. The data shows that Nilo and PFT treatment leads to reduction in the markers of mTOR activation. **f, g** Immunoblot analysis for pAMPK, AMPK, pS555ULK1 and ULK1. The data shows that Nilo and PFT leads to activation of AMPK and ULK1. Quantitative analysis of each figure was presented in bar graph. α-Tub (α-tubulin) was used as a loading control. Data are means ±SEM from *n* = 3. *p < 0.05; **p < 0.01; ***p < 0.001, Student’s *t* test

Upon establishing that c-Abl activation inhibits autophagy in a p53 dependent manner, we asked whether c-Abl and p53 could be targeted to enhance basal autophagy in neuronal cells. As expected, a c-Abl inhibitor, nilotinib (Nilo), induces basal autophagy flux, as indicated by the analysis of bafilomycin A1 (BafA1) dependent the turnover of LC3-II (Fig. 5a). Our results with neuronal cells (CLU and M17 neuroblastoma cells), show that c-Abl inhibition by Nilo treatment leads to significant increase in the basal LC3-II concomitantly deceasing p62 levels compared to control cells (Fig. 5a,b). With the BafA1 treatment (Fig. 5a), there was an expected increase in the LC3-II levels with the greater BafA1-dependent increase in LC3-II with Nilo treatment. This result confirms that Nilo increases autophagy flux. Analysis of autophagy flux at multiple time points following Nilo treatment shows increased autophagy flux at 12 h, but not at 6 h, of Nilo treatment in CLU cells (Additional file 1: Figure S11). Analysis of c-Abl showed that the Nilo was able to decrease the levels of pY412c-Abl by 6 h of treatment (Additional file 1: Figure S12). Thus, inhibition of c-Abl is followed by increased autophagy.

Further, inhibition of p53 by PFT treatment also enhanced autophagy in both CLU and M17 neuronal cell lines (Fig. 5a,b). Autophagy is implicated in the turnover of αS, particularly pS129αS [53, 54]. Thus, we examined Nilo treatment can facilitate the clearance of pathological αS (pS129αS) in M17 neuroblastoma cells expressing A53T mutant human αS (Fig. 5c). Our results show that Nilo treatment leads to obvious decrease in the pS129αS levels while only a modest decrease is seen in the total αS levels (Fig. 5c). Collectively, these results support that inhibition of either c-Abl or p53 can enhance basal autophagy and promote clearance of αS in neuronal cells.

### C-Abl and p53 regulate mTOR, ULK1, and AMPK

To understand the signaling mechanism linking autophagy to c-Abl and p53, we explored whether c-Abl and p53 affect cellular pathways that are known to regulate autophagy. We first examined mTOR signaling as autophagy is negatively regulated by mTOR activation. Analysis of cells expressing c-Abl-PP show that inhibition of autophagy by c-Abl-PP is associated with increased mTOR activity as indicated by increase in phospho-S6 (pS6), a marker of mTOR-dependent activation of ribosomal S6 kinase (Additional file 1: Figure S9). The c-Abl-PP-dependent increases in the levels of pS6 and phosphorylated 4EBP1 (p4EBP1), another major target of mTOR, were significantly attenuated by inhibition of p53, confirming that p53 is required for mTOR activation by c-Abl-PP (Additional file 1: Figure S10).

We also examined whether increase in basal autophagy induced by the inhibition of c-Abl or p53 in cultured neuronal cells is associated with a decrease in mTOR activity. Inhibition of c-Abl or p53 in M17 neuroblastoma cells leads to significant decreases in the levels of pS6 and p4EBP1 (Fig. 5d,e), confirming that the autophagy induced by inhibition of c-Abl or p53 involves reduced mTOR activity. UNC-51 like kinase 1 (ULK1) and adenosine monophosphate-activated kinase (AMPK) are well known upstream regulator of mTOR activity where AMPK1 dependent activation of ULK1 is known to cause mTOR inhibition [55]. Thus, we examined the possibility that c-Abl/p53 regulates mTOR activity by effects on AMPK and ULK1. We found that the inhibition of c-Abl or p53 increases the activating autophosphorylation of AMPK (Fig. 5f,g) and active AMPK in turns facilitates autophagy by phosphorylating ULK1 at Ser_555_ (Fig. 5f,g), leading to ULK1 activation, and by inhibition of mTORC1 (Fig. 5d,e), which inhibits autophagy by ULK1 inactivation [56–58]. Moreover, analysis of the CLU cells at various times following Nilo treatment show that prior to autophagy activation, inhibition of c-Abl at 6 h of Nilo treatment is coincident with the decrease in pS6, indicating that c-Abl inhibition us coincident with the inhibition of mTORC1 signaling (Additional file 1: Figure S12). As with the LC-3 (Additional file 1: Figure S11)**,** down stream effector of of mTOR inhibition, activation of AMPK, seen at 12 h, but not 6 h, of Nilo treatment (Additional file 1: Figure S12). In summary, these data show that Nilo enhances autophagy-lysosomal clearance of αS by modulating c-Abl/p53 pathway through AMPK activation and down regulation of mTORC1 signal pathway.

### Nilotinib treatment increases lifespan of Tg animals, decreases c-Abl and p53 activation, and improves autophagy

**Fig. 6:**
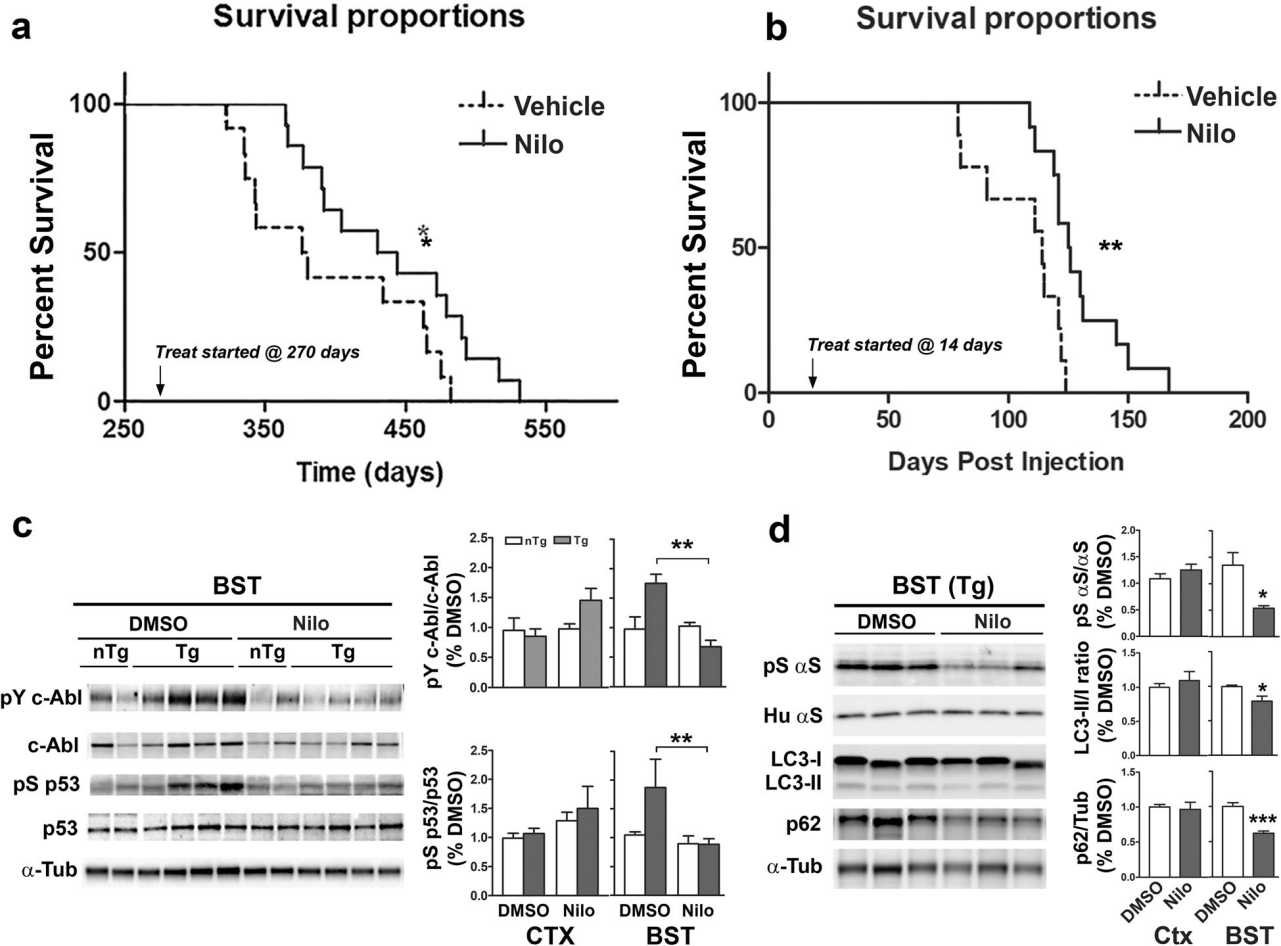
Nilotinib (Nilo) treatment increases lifespan of TgA53T mice, decreases αS pathology, reduces accumulation of p53, and reduces autophagy deficits. **a** Cohorts of TgA53T mice were treated with either Nilo (*n* = 12) or DMSO (Vehicle) (*n* = 12) starting at 9 months of age. Mice were injected intraperitoneally three times weekly until disease onset progressing to end-stage. Nilo treatment significantly increases lifespan in the aged cohort and increased median survival from 378 days in the DMSO group to 437 in the Nilo treated group, *p < 0.05. **b** TgA53T mice were inoculated by IC/IS injection with 3000×g lysate from an end-stage TgA53T mice. Fourteen days following the inoculation, the mice were treated with either Nilo or DMSO (ip, 3X per week). Nilo significantly increased the lifespan in this cohort and increased median survival from 112 days post injection (dpi) for the DMSO treated group to 127 dpi for the Nilo group, **p < 0.01. **c** and **d** Total lysates (TL) of BST from Nilo or DMSO treated mice were immunoblotted for pY412c-Abl, c-Abl, pS15p53 and p53 (**c**) or pS129αS, αS, LC3 and p62 (**d**). The graph shows the quantitative analysis of the immunoblots. *p < 0.05; **p < 0.01; ***p < 0.001, Student’s *t* test. n = 3–6 different animal per group. There was a significant effect for treatment (*p* = 0.021) and interaction (*p* = 0.003) but not species (nTg or Tg, *p* = 0.119) for c-Abl activation by Two-way ANOVA analysis (**c**). A significant effect for treatment (*p* = 0.041) but not species (*p* = 0.143) or interaction (*p* = 0.134) was found for p53 activation by Two-way ANOVA (**c**). A significant effect for treatment (*p* = 0.025) and interaction (*p* = 0.003) for pS129αS level by Two-way ANOVA (**d**). A significant effect for treatment (*p* = 0.01) and interaction (*p* = 0.027) for p62 level by Two-way ANOVA (**d**)

Above studies are consistent with the hypothesis that α-synucleinopathy dependent activation of c-Abl contributes to autophagy defect seen in TgA53T mice. Further, our results are consistent with the prior studies showing that the increased c-Abl activity promotes α-synucleinopathy and the loss of c-Abl expression delays onset of α-synucleinopathy in TgA53T mice [24]. However, because the prior study uses genetic models with life-long alterations in c-Abl expression, translational relevance of the study could be limited. Thus, we tested whether preclinical pharmacological inhibition of c-Abl with Nilo in mature TgA53T model can attenuate the onset of pathology. We used two animal models of α-synucleinopathy to determine if c-Abl inhibition using Nilo, an FDA approved, brain penetrant c-Abl inhibitor [21], can attenuate α-synucleinopathy in vivo (Fig. 6). In the first model, cohorts of TgA53T mice were allowed to age and develop the disease with aging. The Nilo/Vehicle treatments were started at ~ 270 days of age, when approximately 20% of the TgA53T littermates have started to show motor abnormalities, to better approximate therapeutic regimen that might be used in clinics [39] (Fig. 6a). In this model, Nilo treatment significantly increased mean average life span of the TgA53T animals compared to the vehicle treated group, from 378 days to 437 days.

The second model is established by inoculation of TgA53T mice [36] where the pathogenic lysate from end-stage TgA53T mice was injected into the cortex and striatum of pre-symptomatic Tg mice at 6 months of age (Fig. 6b). As expected, inoculation of pathogenic lysates leads to rapid onset of motoric abnormalities between 80 and 100 days post inoculation (dpi) that progress to end-stage within 14–21 days after onset of motoric symptoms (Fig. 6b). In this model, Nilo/Vehicle treatment was initiated at 14 days post injection (dpi). As in the naturally aging model, Nilo significantly increased the mean survival from 112 dpi to 127 dpi in the inoculation model (Fig. 6b).

Biochemical analysis shows that, with the increase in lifespan, Nilo treatment reduced c-Abl activation in brain stem (Fig. 6c). Significantly, Nilo treatment also reduced the levels of active p53 (pS_15_p53) (Fig. 6c), confirming that in α-synucleinopathy, accumulation of active p53 is tightly linked to c-Abl activation. No change in c-Abl or p53 activity was observed in the cortex (Fig. 6c). Furthermore, the levels of pS129αS in the brainstem was significantly decreased in Nilo treated subjects compared the vehicle (DMSO) treated subjects (Fig. 6d), indicating that preclinical inhibition of c-Abl was sufficient to decrease accumulation of αS aggregates. In conjunction with the decrease in pathologic αS, Nilo treatment also reduced the levels of LC3-II and p62 (Fig. 6d), consistent with the idea that c-Abl inhibition is improves autophagic flux.

**Fig. 7:**
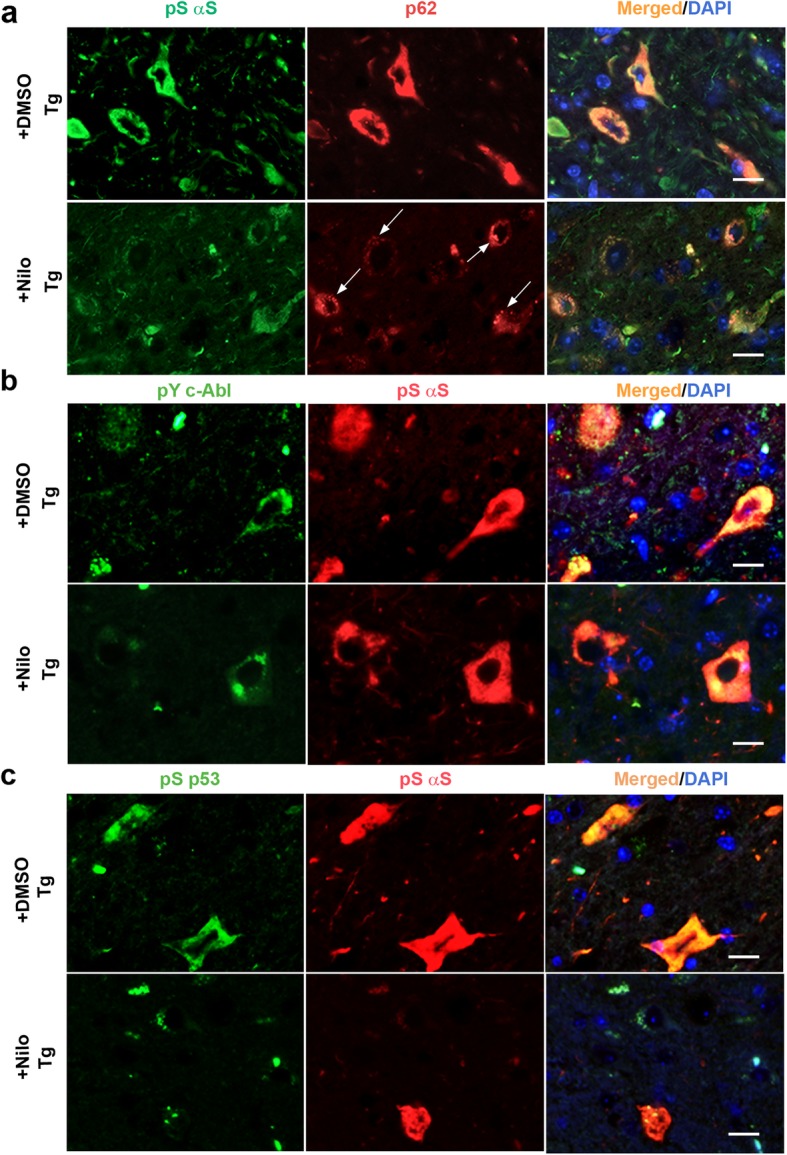
Nilotinib (Nilo) treatment reduces neuronal accumulation of pS129αS, activated c-Abl, activated p53, and p62. Paraffin embedded sagittal brain sections from Nilo and DMSO treated end-stage TgA53T mice were used for immunofluorescence co-localization of pS129αS with p62 (**a**), pY245c-Abl (**b**) and pS15p53 (**c**). Shown are the brainstem regions. The results show that Nilo treatment leads to reduced neuronal accumulation of pS129αS (**a-c**), p62 (**a**), pY245c-Abl (**b**)**,** and pS15p53 (**c**). Scale bar, 20 μm

To further confirm that the therapeutic effects of Nilo in brainstem is linked to mechanistic events outlined using cultured cells, we compared the co-localization of p62, pY245c-Abl and pS15p53 with pS129αS in the brainstem neurons of DMSO (vehicle) and Nilo treated animals (Fig. 7). Both p62 and pS129αS abundantly co-accumulate in TgA53T mice neurons treated with DMSO, whereas Nilo treated animal showed a comparatively lower intensity of p62 and pS129αS immunoreactivity (Fig. 7a). Further, p62 in the Nilo treated neurons appear as well-defined punctate structures (Fig. 7a, arrows), suggesting that the p62 inclusions might be cargos inside autophagosome targeted for autophagic clearance. Similarly, Nilo treatment lead to overall reducing in the c-Abl and p53 immunoreactivity associated with pS129αS positive neurons (Fig. 7b,c). Overall, our data supports the hypothesis that c-Abl inhibition by Nilo promotes clearance of toxic αS via activation of autophagy.

**Fig. 8:**
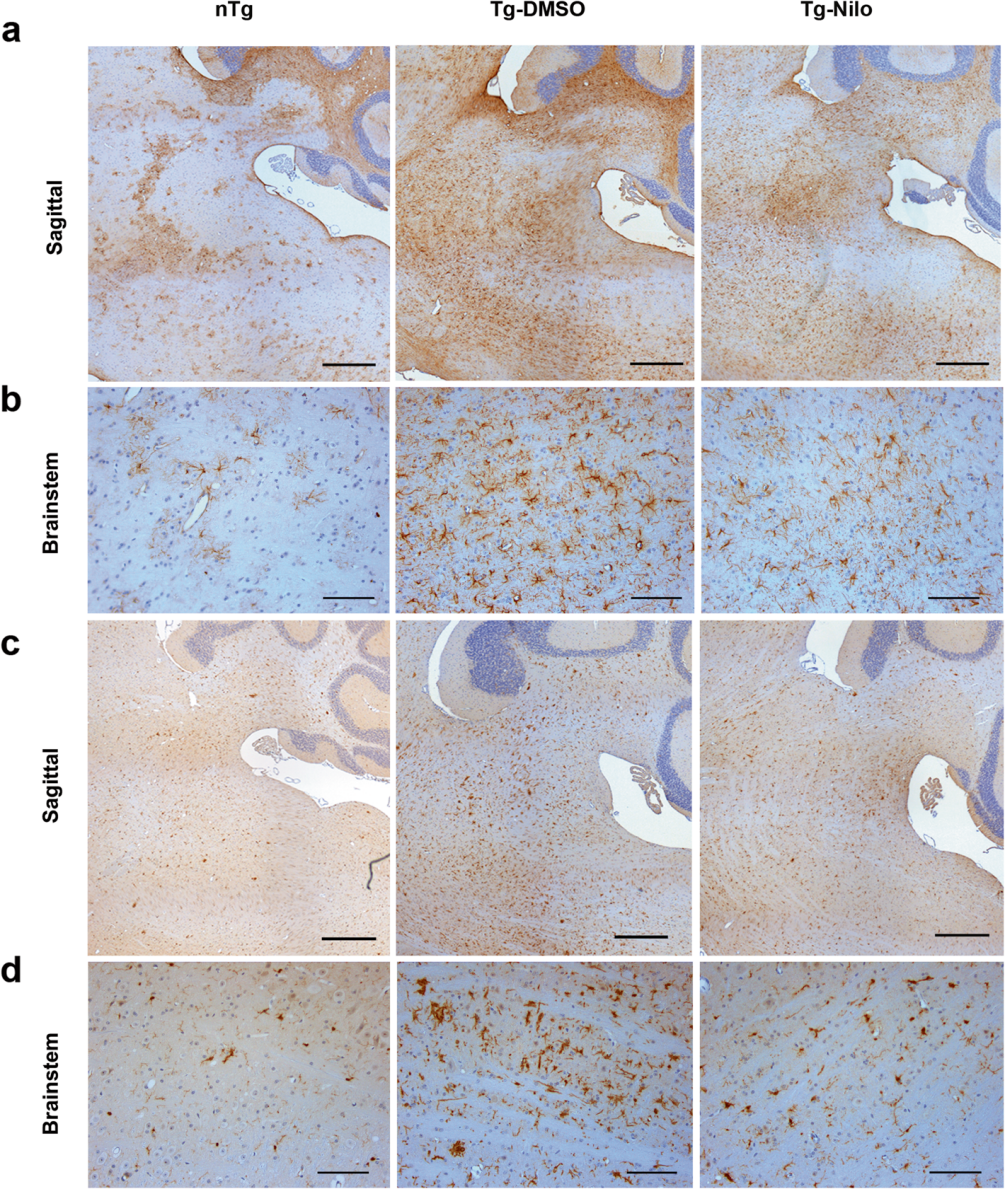
Nilotinib treatment decreases astrocyte and microglia reactivity in the brain. Nilotinib (Nilo) or DMSO (vehicle) treated end-stage TgA53T (Tg) mice and aged-match littermate nTg were used to obtain brain tissue and immunostained using GFAP antibody (**a, b**) and Iba1 antibody (**c, d**). Tg-DMSO shows an increase of highly active and proliferated astrocytes compare to nTg control, whereas Nilo treatment shows a reduction of astrocytes and microglia reactivity and proliferation compare to vehicle (Tg-DMSO) treatment. **a, c** Stitched-image of nTg, Tg-DMSO and Tg-Nilo of sagittal brain. Scale bar, 500 μm. **b, d** Brainstem. Scale bar, 100 μm

Above results clearly indicate that Nilo treatment activates autophagy, reduces αS pathology, and attenuates neurodegeneration. To provide independent determinations that Nilo treatment was associated with reduced neurodegeneration, we examined the activation of astrocytes and microglia, two common indicators of neurodegeneration (Fig. 8**;** Additional file 1: Figure S13). Immunocytochemical analysis for astrocytes using anti-GFAP antibody (Fig. 8a,b**;** Additional file 1: Figure S13a) and microglia using anti-IbaI antibody (Fig. 8c,d; Additional file 1: Figure S13b) shows that obvious increase in GFAP and IbaI immunoreactivity in TgA53T mice compared to nTg littermates. Significantly, Nilo treatment of TgA53T mice leads to attenuation of both GFAP and IbaI immunoreactivity (Fig. 8**;** Additional file 1: Figure S13), confirming that Nilo treatment attenuates neurodegeneration.

While Nilo treatment leads to dramatic reduction in α-synucleinopathy-related abnormalities in brainstem, analysis of spinal cord reveals that Nilo was not able to reduce the levels of active c-Abl or reduce αS pathology, including accumulation of pS129αS, in spinal cord (Additional file 1: Figures S14 and S15). Further, Nilo treatment did not alter autophagy markers, LC3-II and p62, in the spinal cord of TgA53T mice (Additional file 1: Figure S15)**.** Finally, Nilo treatment did not reduce activation of astrocytes and microglia in TgA53T spinal cord (Additional file 1: Figure S16). The lack of Nilo to inhibit c-Abl and provide neuroprotection in spinal cord accounts for the modest therapeutic effect of Nilo, where the disease onset is delayed and not prevented. Significantly, the therapeutic effects seen with Nilo treatments are comparable to that achieved with the constitutive brain c-Abl-knockout mice [24]. Because, only the brain stem was analyzed for pathology in the c-Abl-knockout study [24], the status of neuropathology in the spinal cord is unknown. Regardless, in addition to the resistance of spinal cord c-Abl to inhibition by Nilo, it is possible that c-Abl-independent processes, such as chronic endoplasmic reticulum stress [39, 40], contribute to neurodegeneration in spinal cord neurons.

**Fig. 9:**
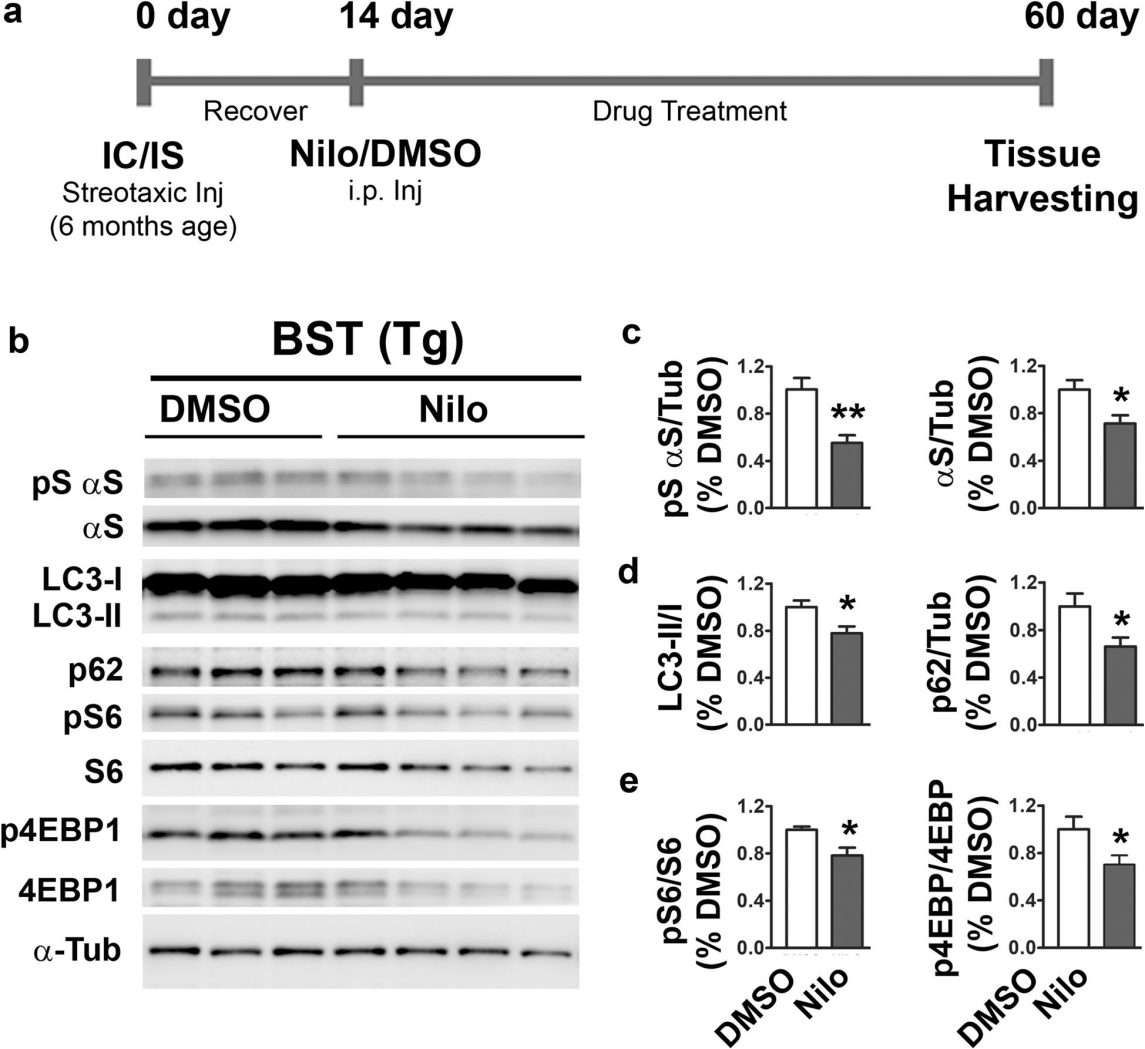
Modulation of mTOR signaling by nilotinib (Nilo) treatment in vivo. Six-month-old TgA53T mice were subjected to IC/IS injection with 3000×g brainstem/spinal cord lysates from end stage mice as described in “Materials and Methods”. Fourteen days post IC/IS injection, Nilo or DMSO were administered for 45 days (i.p. 3× per week) prior to harvesting of the brain regions. **a** A brief experimental time line. **b** Total lysates from the brainstem (BST) of experimental subjects were immunoblotted for pS129αS, αS, LC3, p62, pS6, S6, p4EBP1, 4EBP1 and α-Tub. Quantitative analysis for pS129αS/αS (**c**), LC3/p62 (**d**), pS6/p4EBP1 (**e**) shows that Nilo treatment reduces αS pathology and decreases mTOR activity at presymptomatic stages. All values represent mean ± SEM, n = 3–4, *p < 0.05; **p < 0.01, Student’s *t* test

We also examined whether Nilo treatment was delaying the progression of α-synucleinopathy by enhancing autophagy during the presymptomatic stage of the disease. For this study, we evaluated the autophagy related parameters at 60 dpi of toxic lysates inoculated into IC/IS, well prior to disease onset at ~ 100 dpi. The cohorts of mice were treated with DMSO or Nilo from 14 to 60 dpi (Fig. 9a). At this time point, our analysis shows that αS pathology is starting to appear (data not shown), but the mice have yet to develop any clinical symptoms. Our analysis shows that even at this early stage, both pS129αS and total αS levels were significantly decrease by Nilo treatment (Fig. 9b,c). Consistent with the improved autophagic flux (Fig. 6), Nilo significantly decreased LC3-II and p62 levels at the presymptomatic stages (Fig. 9b, d). We also found that Nilo leads to inhibition of mTORC1, indicated by significant reductions in the levels of S6 and 4EBP1 phosphorylation (Fig. 9b,e). Collectively, our data indicate that Nilo can induce autophagy via mTOR inhibition at early stages of α-synucleinopathy.

## Discussion

Current study investigated the pathological connection between non-receptor tyrosine kinase c-Abl and α-synucleinopathy. Increased c-Abl activity has been found in many neurodegenerative diseases and in PD, c-Abl activation in sporadic PD has implicated parkin, an E3 ubiquitin ligase associated with autosomal recessive PD, as potential pathogenic target [13, 19]. However, recent studies indicate additional pathogenic target of c-Abl. For example, cAbl has been shown to phosphorylate αS [27], potentially leading to increase αS aggregation [24] and c-Abl inhibition promotes αS clearance via activation of autophagy [23]. This study investigates an alternate pathway where c-Abl activation inactivates Mdm2, leading to the accumulation of p53 in the cytoplasm and impaired autophagy. We show that activation of c-Abl in α-synucleinopathy is associated with increased activation of p53. We also show that c-Abl-dependent inhibition of autophagy requires p53 activity and the c-Abl/p53 activity is associated with mTOR signaling. Further, inhibition of c-Abl kinase by nilotinib (Nilo), an FDA approved inhibitor of c-Abl, is coined with increasing AMPK/ULK1 activity to enhance autophagy clearance of pathologic αS. Finally, we show that Nilo treatment of TgA53T mouse model delays onset of clinical symptoms and facilitate autophagic clearance of pathological αS. Overall, our results support the novel conclusion that α-synucleinopathy-dependent activation of c-Abl/p53 is a key underlying cause of autophagy deficit in α-synucleinopathy and that c-Abl/p53 activity is a significant target for therapeutic intervention in α-synucleinopathies.

### C-Abl/Mdm2/p53 pathway and its implication in α-synucleinopathy

Many studies have established the c-Abl/Mdm2/p53 pathway [16, 59–62]. c-Abl is known to interact with both Mdm2 and p53 [16, 60]. Mdm2 antagonizes p53 by ubiquitinating p53 and targeting p53 for the proteasomal degradation, as well as binding to the N-terminus of p53 to prevent its transcriptional activity [60]. Stabilization of p53 in response to cellular stress is due to a decreased Mdm2 levels and/or interactions between p53 and Mdm2 [46, 63]. Active c-Abl is known to interact with Mdm2 and tyrosine-phosphorylation of Mdm2 by c-Abl decreases its ability to degrade p53 [30, 60]. We extend these studies in cancer cells to show that in neuronal cells and in brain, αS pathology is associated with activation of c-Abl, decrease in Mdm2/Hdm2 levels, and increased p53 activity.

### Activation of p53 involved in PD pathology and cytosolic p53 impairs autophagic clearance

Many studies on neurodegenerative diseases, including PD, show an increase in p53 activation [64]. Expression of p53 is increased in the diseased tissue of PD patients is also elevated in the substantia nigra neurons [52, 65, 66]. Neuronal populations undergoing apoptosis with abnormal cytosolic accumulation of p53 were previously found in this A53TαS Tg mouse model [31]. In this study, we show that the accumulation of active c-Abl and cytosolic p53 selectively occurs in neurons with αS pathology, supporting that αS pathology induces c-Abl activation and subsequent activation of p53. While p53 activation is normally associated with increased autophagy, via transcriptional induction of autophagy genes [67], Tasdemir et al. showed that the accumulation of cytosolic p53, but not nuclear p53, impairs autophagy [33, 68]. Recent study also shows that inhibition of p53 promotes parkin-mediated mitophagy in pancreatic beta cells [69]. Thus, our results are consistent with these previous studies and provide mechanistic basis for how c-Abl activation leads to autophagy deficits in α-synucleinopathies. In this regard, studies have shown that mitochondrial toxicity is inhibited by pifithrin-α independent of p53 [70], indicating that pifithrin-α has other cellular targets. However, our results with pifithrin-α is likely to be p53 dependent as the p53 gene silencing was sufficient to reverse autophagy deficit caused by constitutively active c-Abl (Figs. 4 and 5).

In addition to the c-Abl-dependent p53 activation and autophagy deficits, a series of studies implicate c-Abl as a direct regulator of αS. Mahul-Mellier et al., (2014) showed that c-Abl directly interacts with αS and catalyze αS phosphorylation, mainly at tyrosine 39 (Y39) and, to lesser extent, at tyrosine 125 (Y125) [27]. A recent study [24] shows that constitutively increasing c-Abl activity in brain promotes αS pathology and the constitutive loss of c-Abl expression in brain attenuates αS pathology. This study implicates c-Abl in increasing αS aggregation by αS phosphorylation at Y39 (pY39αS) [24]. However, the role of pY39αS in promoting αS aggregation can be debated as biochemical and biophysical studies show that pY39αS, while more toxic to cells, aggregated much more slowly than the wild type of αS [29]. Therefore, it is unclear that increase in pY39αS level is sufficient to cause increased αS aggregation in cells. Rather, it is more likely that c-Abl dependent autophagy deficit, leading to accumulation of αS, is a more likely contributor to the increased αS pathology observed in neurons with increased c-Abl activity. Thus, our hypothesis would be able to incorporate increased αS aggregation as well as increase in the levels of toxic soluble oligomers from pY39αS.

### Pharmacological inhibition of c-Abl using Nilotinib modulates autophagy and attenuates α-synucleinopathy in TgA53T model

Studies indicate that c-Abl might be a promising therapeutic target for α-synucleinopathy [24, 25, 45, 71] and a recent study demonstrated that a pharmacological c-Abl inhibitor, nilotinib (Nilo) enable to cross blood brain barrier [72, 73]. The previous c-Abl inhibition study [25] showed that Nilo treatment of A53T transgenic mice starting at early ages (6–8 months old) lead to reduced levels of total αS levels in the forebrain regions but the effects of Nilo on overt αS pathology (pS129αS) and the disease onset was not reported. While constitutive loss of c-Abl expression in brain can increase the life span in TgA53T model [24], it is unclear if the attenuation of c-Abl activity at older ages, corresponding to preclinical stages of disease, has therapeutic benefit. In the current study, we show that pharmacological inhibition of c-Abl by Nilo in aged, preclinical stage TgA53T mice leads to modest but significant delay in disease onset. Further, we also show that Nilo treatment following inoculation of toxic lysate also delays disease onset, supporting the notion that c-Abl inhibition is able to slow disease initiation and disease progression. Further, we show using cell culture studies as well as biochemical analysis of mouse tissues that c-Abl activation inhibits autophagy in a p53-activity dependent manner. We also show that inhibition of c-Abl by Nilo decreases p53 levels and increase autophagy. More important, we show that Nilo increases autophagy in vitro and in vivo by activating AMPK phosphorylation and decreasing mTOR activation. Our previous study also shows that salubrinal, an anti-endoplasmic reticulum (ER) stress compound, significantly attenuates αS disease manifestation [39]. In the present study, the neuroprotective effect of Nilo may also have a contribution in anti-ER therapeutic intervention like salubrinal.

We also propose that the mechanism outlined here might be generally applicable to other age-related neurodegenerative diseases. Proteostatic imbalance, including autophagy impairment, is mechanistically implicated in the pathogenesis of multiple neurodegenerative diseases. Thus, we hypothesize that similar oxidative stress-induced c-Abl activation in other neurodegenerative diseases will also cause autophagy deficits. For example, c-Abl pathway is described as a potential therapeutic target in amyotrophic lateral sclerosis (ALS) [74], AD and PD [75]. Therefore, the c-Abl activation could be a general molecular defect for age-related neurodegeneration.

## Conclusions

In conclusion, our findings using in vivo and in vitro models demonstrated that αS pathology induced c-Abl activation leads to p53-depdendent mTOR activation and autophagy impairment. The pathological relevance of the pathway is shown by the fact that Nilo treatment delays disease in TgA53T mouse model of α-synucleinopathy and ameliorate signs of autophagic deficits. Thus, we uncovered a previously unknown pathological mechanism linking with c-Abl/p53 activation, mTOR/AMPK/ULK1, and autophagy. Additional studies to define further the mechanistic details will facilitate deeper understanding of pathogenic events in neurodegenerative disease and lead to novel therapeutic approaches.

## Supplementary information

**Additional file 1: Figure S1.** Increased 3-nitrotyrosine (3-NT) immunoreactivity in TgA53T neurons indicate neuronal oxidative stress. **Figure S2.** Activation of c-Abl and p53 is associated with α-synucleinopathy. **Figure S3.** Activation of Mdm2 in A53TαSyn Tg Mice brainstem (BST). **Figure S4.** Increased cytosolic p53 in TgA53T neurons. **Figure S5.** Colocalization of p62 accumulation with pS129αS in TgA53T. **Figure S6.** c-Abl activity and autophagy in HEK-293 cells. **Figure S7.** c-Abl activity modulates nutrient starvation induced autophagy. **Figure S8.** Constitutively active c-Abl suppresses Mdm2 levels and increases p53 levels. **Figure S9.** Increased mTOR activation with c-Abl activation. **Figure S10.** c-Abl-dependent effects on mTOR and AMPK is blocked by inhibition of p53. **Figure S11.** Inhibition of c-Abl by nilotinib (Nilo) modulate basal autophagy, in neuronal cells. **Figure S12.** c-Abl-dependent effects on mTOR and AMPK. **Figure S13.** Nilotinib treatment decreases activation of astrocyte and microglia in the TgA53T Cerebellum and Brainstem. **Figure S14.** Nilotinib treatment is not able to inhibit c-Abl in the TgA53T spinal cord. **Figure S15.** Nilotinib treatment is not able to change αS pathology or attenuates autophagy deficit in spinal cord. **Figure S16.** Nilotinib treatment is not able to inhibit astrocyte/microglia activation in spinal cord. **Table S1.** Antibodies used for Western blotting and immunohistochemistry.

## Data Availability

The data and materials are available from corresponding author on reasonable request.

## Abbreviations

3-NT: 3-nitrotyrosine
4EBP1: Eukaryotic translation initiation factor 4E (eIF4E)-binding protein 1
Akt (PKB): protein kinase B
AMPK: adenosine monophosphate-activated protein kinase
Baf A1: bafilomycin A1
BST: Brainstem
CTX: Cortex
DMEM: Dulbecco’s modified Eagle’s medium
DMSO: Dimethyl sulfoxide
EBSS: Earle’s balanced salt solution
EV: Empty vector
GAPDH: glyceraldehyde 3-phosphate dehydrogenase
GFAP: Glial fibrillary acidic protein
Iba1: Ionized calcium binding adaptor molecule 1
LC3: microtubule-associated protein 1A/1B-light chain 3
Mdm2: Mouse double minute 2 homolog
mTOR: mammalian target of rapamycin
NeuN: Hexaribonucleotide Binding Protein-3
Nilo: Nilotinib
p62 (SQSTM1): sequestosome 1
PFT: Pifithrin-α
S6: ribosomal protein S6
SPC: Spinal Cord
ULK1: unc-51 like autophagy activating kinase 1
αS: α-synuclein
