# Supplementary figures and images for: α-Synucleinopathy associated c-Abl activation causes p53-dependent autophagy impairment
Source: Mol Neurodegener. 2020 Apr 16;15:27. doi: 10.1186/s13024-020-00364-w (PMC7164361; doi:10.1186/s13024-020-00364-w)

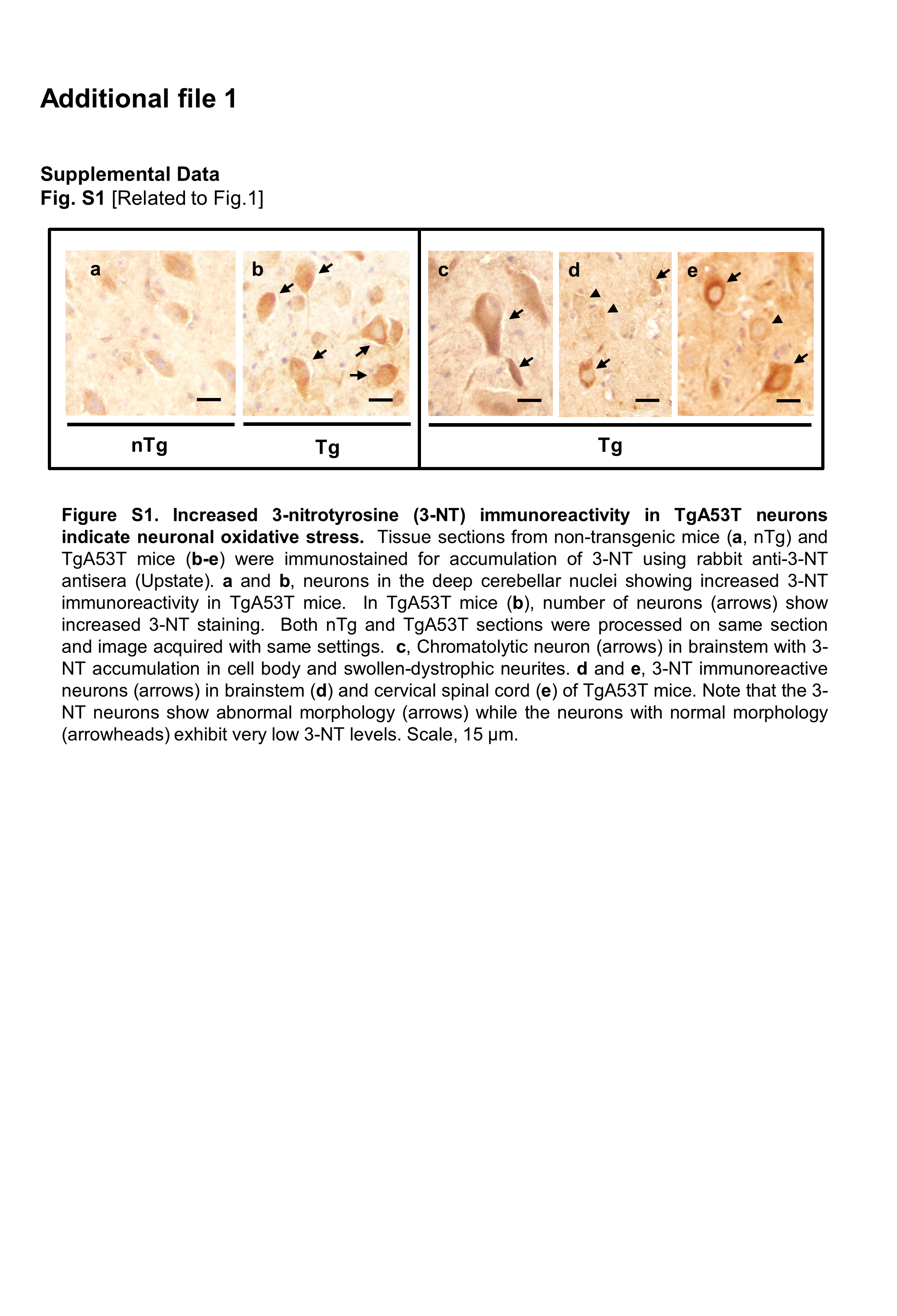

Supplement: Supplementary file 1 — Additional file 1: Figure S1. Increased 3-nitrotyrosine (3-NT) immunoreactivity in TgA53T neurons indicate neuronal oxidative stress. Figure S2. Activation of c-Abl and p53 is associated with α-synucleinopathy. Figure S3. Activation of Mdm2 in A53TαSyn Tg Mice brainstem (BST). Figure S4. Increased cytosolic p53 in TgA53T neurons. Figure S5. Colocalization of p62 accumulation with pS129αS in TgA53T. Figure S6. c-Abl activity and autophagy in HEK-293 cells. Figure S7. c-Abl activity modulates nutrient starvation induced autophagy. Figure S8. Constitutively active c-Abl suppresses Mdm2 levels and increases p53 levels. Figure S9. Increased mTOR activation with c-Abl activation. Figure S10. c-Abl-dependent effects on mTOR and AMPK is blocked by inhibition of p53. Figure S11. Inhibition of c-Abl by nilotinib (Nilo) modulate basal autophagy, in neuronal cells. Figure S12. c-Abl-dependent effects on mTOR and AMPK. Figure S13. Nilotinib treatment decreases activation of astrocyte and microglia in the TgA53T Cerebellum and Brainstem. Figure S14. Nilotinib treatment is not able to inhibit c-Abl in the TgA53T spinal cord. Figure S15. Nilotinib treatment is not able to change αS pathology or attenuates autophagy deficit in spinal cord. Figure S16. Nilotinib treatment is not able to inhibit astrocyte/microglia activation in spinal cord. Table S1. Antibodies used for Western blotting and immunohistochemistry. [file 13024_2020_364_MOESM1_ESM.zip › 13024_2020_364_MOESM1_ESM/Figure S1.TIF]

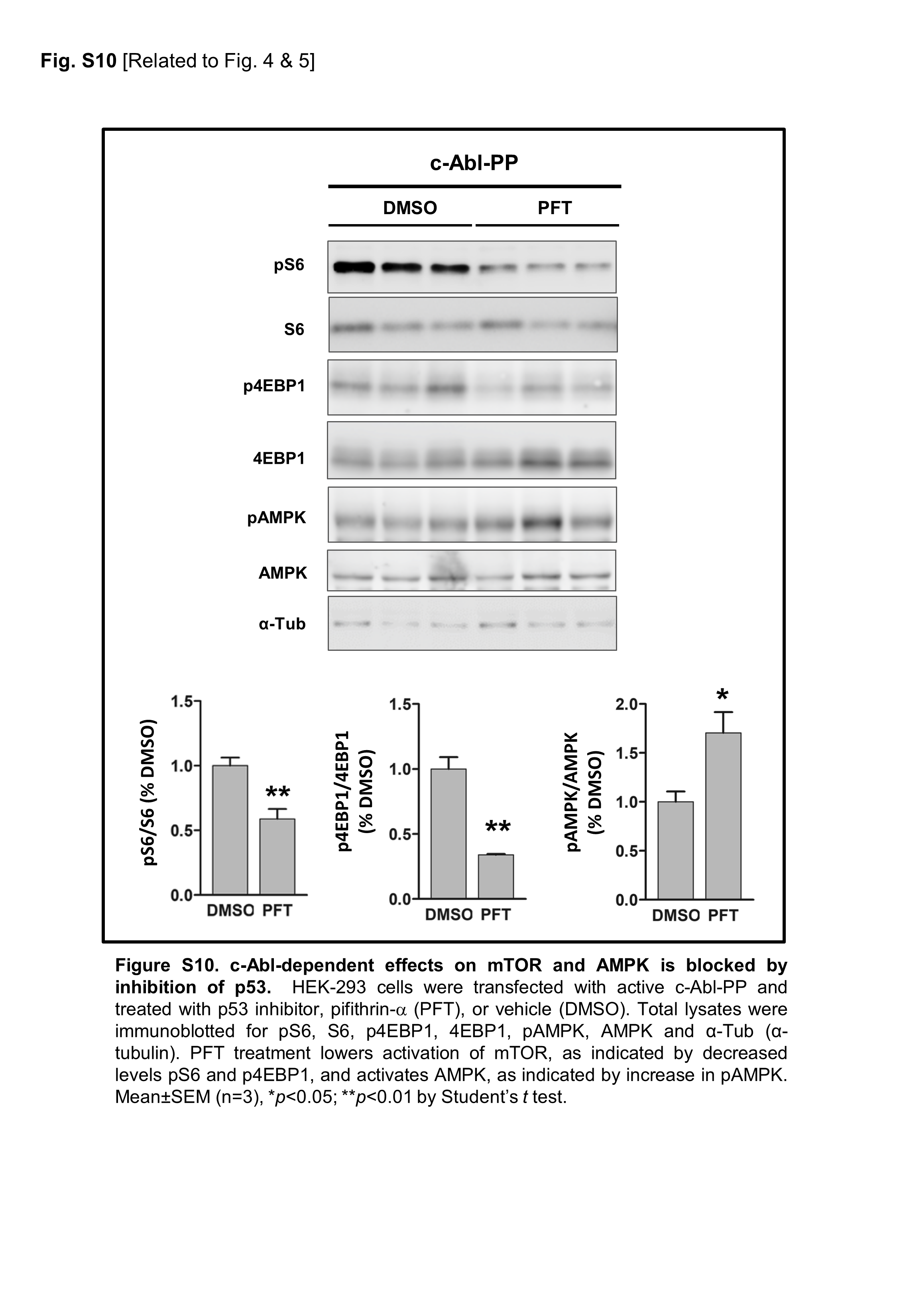

Supplement: Supplementary file 1 — Additional file 1: Figure S1. Increased 3-nitrotyrosine (3-NT) immunoreactivity in TgA53T neurons indicate neuronal oxidative stress. Figure S2. Activation of c-Abl and p53 is associated with α-synucleinopathy. Figure S3. Activation of Mdm2 in A53TαSyn Tg Mice brainstem (BST). Figure S4. Increased cytosolic p53 in TgA53T neurons. Figure S5. Colocalization of p62 accumulation with pS129αS in TgA53T. Figure S6. c-Abl activity and autophagy in HEK-293 cells. Figure S7. c-Abl activity modulates nutrient starvation induced autophagy. Figure S8. Constitutively active c-Abl suppresses Mdm2 levels and increases p53 levels. Figure S9. Increased mTOR activation with c-Abl activation. Figure S10. c-Abl-dependent effects on mTOR and AMPK is blocked by inhibition of p53. Figure S11. Inhibition of c-Abl by nilotinib (Nilo) modulate basal autophagy, in neuronal cells. Figure S12. c-Abl-dependent effects on mTOR and AMPK. Figure S13. Nilotinib treatment decreases activation of astrocyte and microglia in the TgA53T Cerebellum and Brainstem. Figure S14. Nilotinib treatment is not able to inhibit c-Abl in the TgA53T spinal cord. Figure S15. Nilotinib treatment is not able to change αS pathology or attenuates autophagy deficit in spinal cord. Figure S16. Nilotinib treatment is not able to inhibit astrocyte/microglia activation in spinal cord. Table S1. Antibodies used for Western blotting and immunohistochemistry. [file 13024_2020_364_MOESM1_ESM.zip › 13024_2020_364_MOESM1_ESM/Figure S10.TIF]

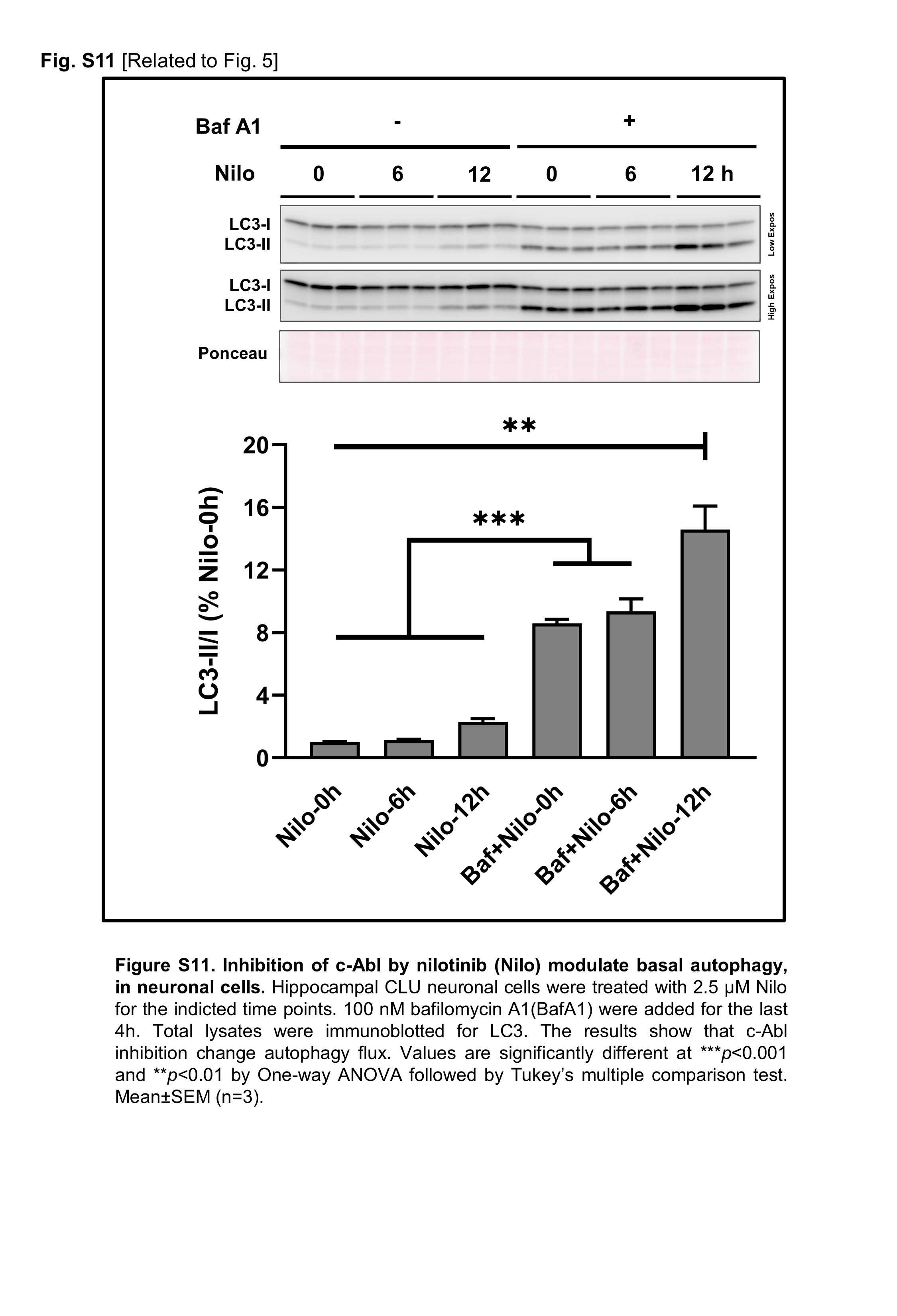

Supplement: Supplementary file 1 — Additional file 1: Figure S1. Increased 3-nitrotyrosine (3-NT) immunoreactivity in TgA53T neurons indicate neuronal oxidative stress. Figure S2. Activation of c-Abl and p53 is associated with α-synucleinopathy. Figure S3. Activation of Mdm2 in A53TαSyn Tg Mice brainstem (BST). Figure S4. Increased cytosolic p53 in TgA53T neurons. Figure S5. Colocalization of p62 accumulation with pS129αS in TgA53T. Figure S6. c-Abl activity and autophagy in HEK-293 cells. Figure S7. c-Abl activity modulates nutrient starvation induced autophagy. Figure S8. Constitutively active c-Abl suppresses Mdm2 levels and increases p53 levels. Figure S9. Increased mTOR activation with c-Abl activation. Figure S10. c-Abl-dependent effects on mTOR and AMPK is blocked by inhibition of p53. Figure S11. Inhibition of c-Abl by nilotinib (Nilo) modulate basal autophagy, in neuronal cells. Figure S12. c-Abl-dependent effects on mTOR and AMPK. Figure S13. Nilotinib treatment decreases activation of astrocyte and microglia in the TgA53T Cerebellum and Brainstem. Figure S14. Nilotinib treatment is not able to inhibit c-Abl in the TgA53T spinal cord. Figure S15. Nilotinib treatment is not able to change αS pathology or attenuates autophagy deficit in spinal cord. Figure S16. Nilotinib treatment is not able to inhibit astrocyte/microglia activation in spinal cord. Table S1. Antibodies used for Western blotting and immunohistochemistry. [file 13024_2020_364_MOESM1_ESM.zip › 13024_2020_364_MOESM1_ESM/Figure S11.TIF]

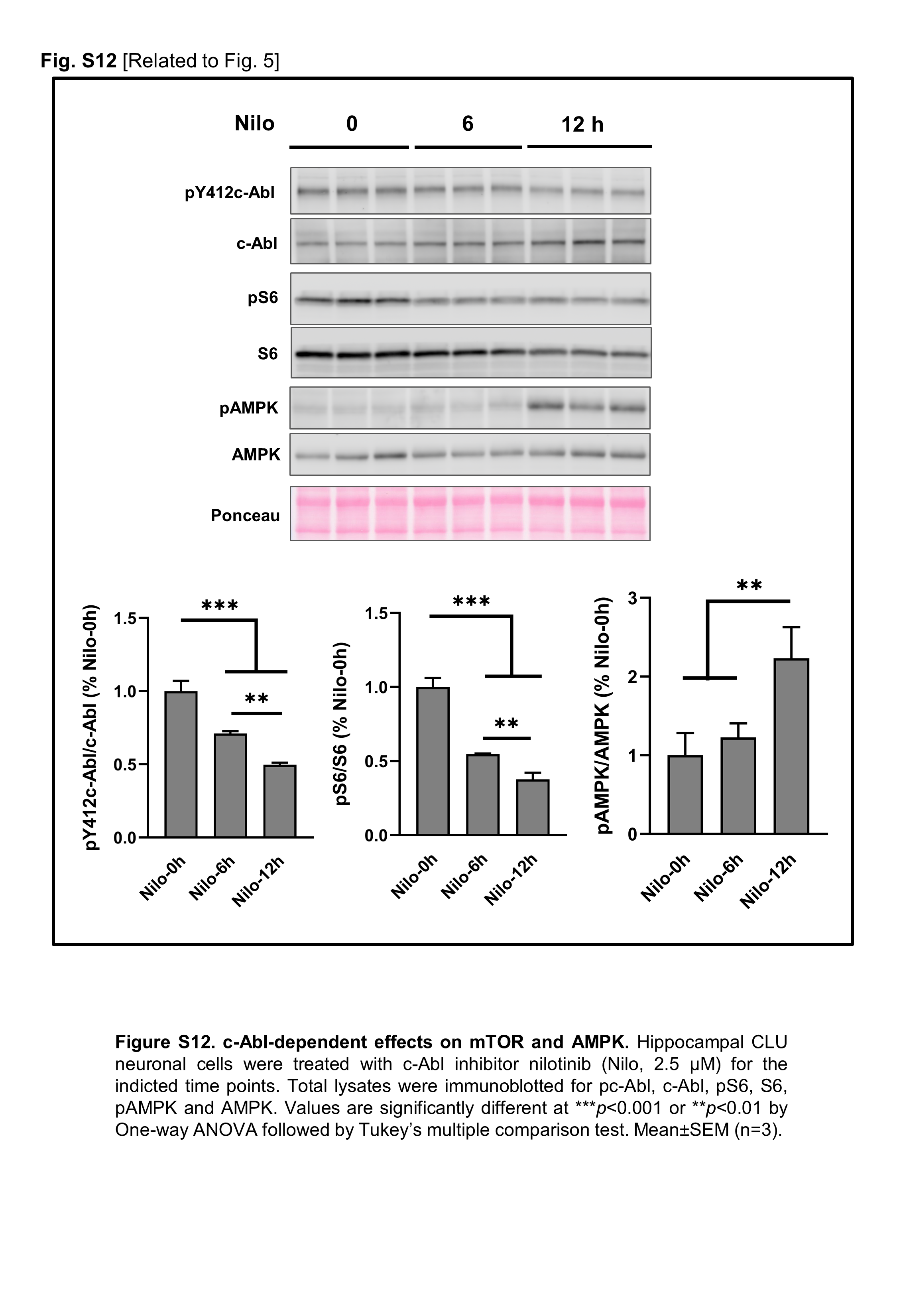

Supplement: Supplementary file 1 — Additional file 1: Figure S1. Increased 3-nitrotyrosine (3-NT) immunoreactivity in TgA53T neurons indicate neuronal oxidative stress. Figure S2. Activation of c-Abl and p53 is associated with α-synucleinopathy. Figure S3. Activation of Mdm2 in A53TαSyn Tg Mice brainstem (BST). Figure S4. Increased cytosolic p53 in TgA53T neurons. Figure S5. Colocalization of p62 accumulation with pS129αS in TgA53T. Figure S6. c-Abl activity and autophagy in HEK-293 cells. Figure S7. c-Abl activity modulates nutrient starvation induced autophagy. Figure S8. Constitutively active c-Abl suppresses Mdm2 levels and increases p53 levels. Figure S9. Increased mTOR activation with c-Abl activation. Figure S10. c-Abl-dependent effects on mTOR and AMPK is blocked by inhibition of p53. Figure S11. Inhibition of c-Abl by nilotinib (Nilo) modulate basal autophagy, in neuronal cells. Figure S12. c-Abl-dependent effects on mTOR and AMPK. Figure S13. Nilotinib treatment decreases activation of astrocyte and microglia in the TgA53T Cerebellum and Brainstem. Figure S14. Nilotinib treatment is not able to inhibit c-Abl in the TgA53T spinal cord. Figure S15. Nilotinib treatment is not able to change αS pathology or attenuates autophagy deficit in spinal cord. Figure S16. Nilotinib treatment is not able to inhibit astrocyte/microglia activation in spinal cord. Table S1. Antibodies used for Western blotting and immunohistochemistry. [file 13024_2020_364_MOESM1_ESM.zip › 13024_2020_364_MOESM1_ESM/Figure S12.TIF]

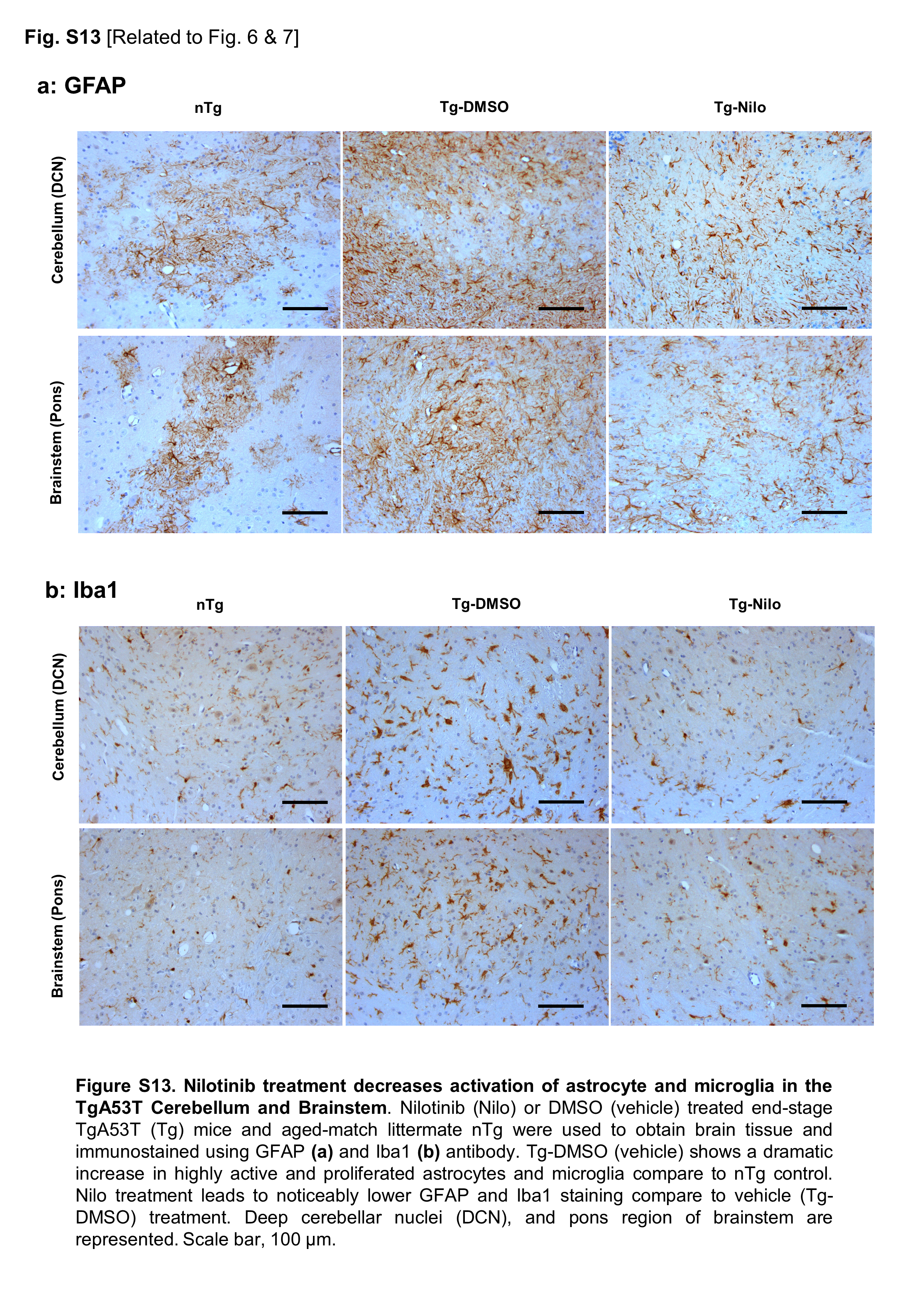

Supplement: Supplementary file 1 — Additional file 1: Figure S1. Increased 3-nitrotyrosine (3-NT) immunoreactivity in TgA53T neurons indicate neuronal oxidative stress. Figure S2. Activation of c-Abl and p53 is associated with α-synucleinopathy. Figure S3. Activation of Mdm2 in A53TαSyn Tg Mice brainstem (BST). Figure S4. Increased cytosolic p53 in TgA53T neurons. Figure S5. Colocalization of p62 accumulation with pS129αS in TgA53T. Figure S6. c-Abl activity and autophagy in HEK-293 cells. Figure S7. c-Abl activity modulates nutrient starvation induced autophagy. Figure S8. Constitutively active c-Abl suppresses Mdm2 levels and increases p53 levels. Figure S9. Increased mTOR activation with c-Abl activation. Figure S10. c-Abl-dependent effects on mTOR and AMPK is blocked by inhibition of p53. Figure S11. Inhibition of c-Abl by nilotinib (Nilo) modulate basal autophagy, in neuronal cells. Figure S12. c-Abl-dependent effects on mTOR and AMPK. Figure S13. Nilotinib treatment decreases activation of astrocyte and microglia in the TgA53T Cerebellum and Brainstem. Figure S14. Nilotinib treatment is not able to inhibit c-Abl in the TgA53T spinal cord. Figure S15. Nilotinib treatment is not able to change αS pathology or attenuates autophagy deficit in spinal cord. Figure S16. Nilotinib treatment is not able to inhibit astrocyte/microglia activation in spinal cord. Table S1. Antibodies used for Western blotting and immunohistochemistry. [file 13024_2020_364_MOESM1_ESM.zip › 13024_2020_364_MOESM1_ESM/Figure S13.TIF]

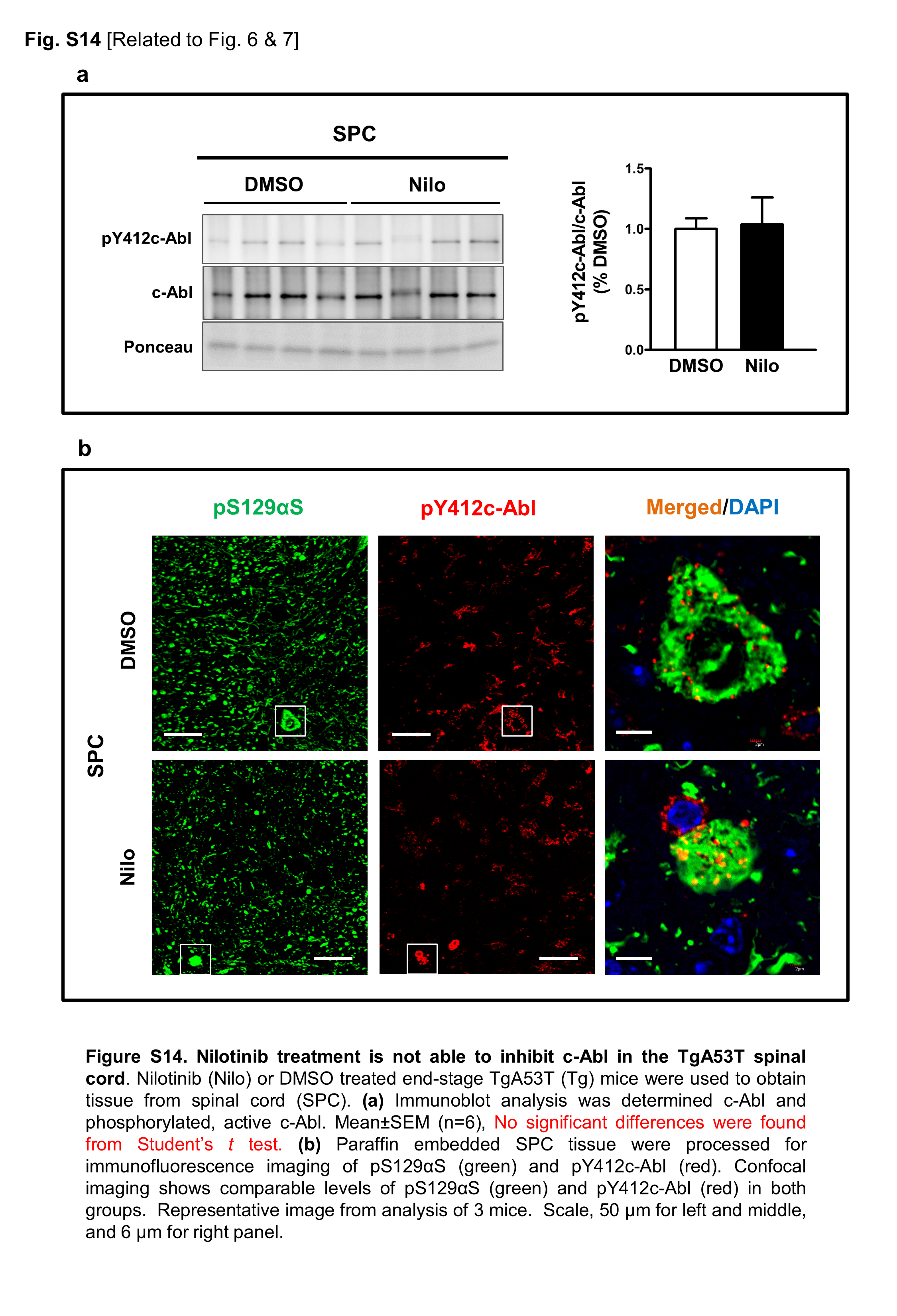

Supplement: Supplementary file 1 — Additional file 1: Figure S1. Increased 3-nitrotyrosine (3-NT) immunoreactivity in TgA53T neurons indicate neuronal oxidative stress. Figure S2. Activation of c-Abl and p53 is associated with α-synucleinopathy. Figure S3. Activation of Mdm2 in A53TαSyn Tg Mice brainstem (BST). Figure S4. Increased cytosolic p53 in TgA53T neurons. Figure S5. Colocalization of p62 accumulation with pS129αS in TgA53T. Figure S6. c-Abl activity and autophagy in HEK-293 cells. Figure S7. c-Abl activity modulates nutrient starvation induced autophagy. Figure S8. Constitutively active c-Abl suppresses Mdm2 levels and increases p53 levels. Figure S9. Increased mTOR activation with c-Abl activation. Figure S10. c-Abl-dependent effects on mTOR and AMPK is blocked by inhibition of p53. Figure S11. Inhibition of c-Abl by nilotinib (Nilo) modulate basal autophagy, in neuronal cells. Figure S12. c-Abl-dependent effects on mTOR and AMPK. Figure S13. Nilotinib treatment decreases activation of astrocyte and microglia in the TgA53T Cerebellum and Brainstem. Figure S14. Nilotinib treatment is not able to inhibit c-Abl in the TgA53T spinal cord. Figure S15. Nilotinib treatment is not able to change αS pathology or attenuates autophagy deficit in spinal cord. Figure S16. Nilotinib treatment is not able to inhibit astrocyte/microglia activation in spinal cord. Table S1. Antibodies used for Western blotting and immunohistochemistry. [file 13024_2020_364_MOESM1_ESM.zip › 13024_2020_364_MOESM1_ESM/Figure S14.TIF]

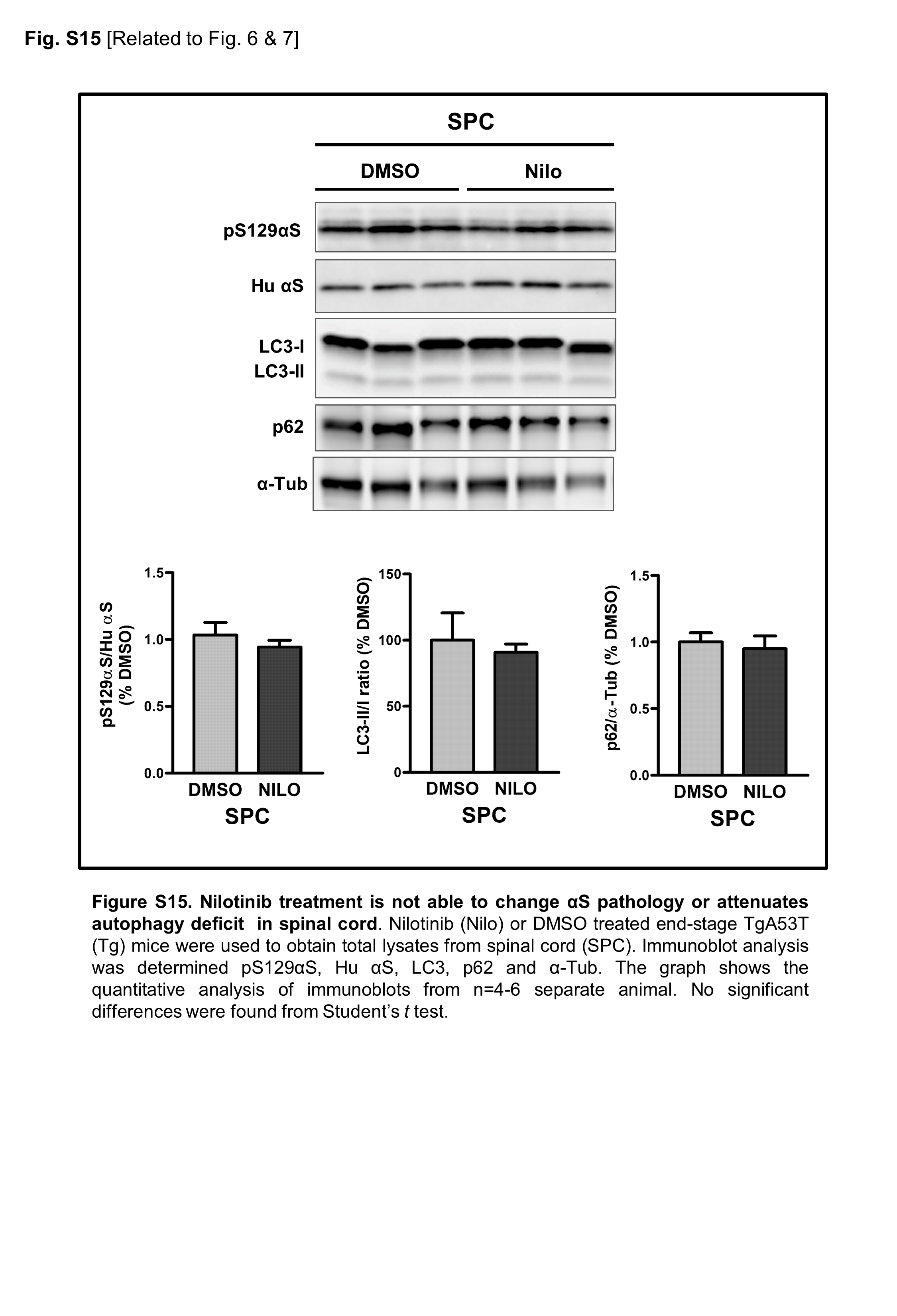

Supplement: Supplementary file 1 — Additional file 1: Figure S1. Increased 3-nitrotyrosine (3-NT) immunoreactivity in TgA53T neurons indicate neuronal oxidative stress. Figure S2. Activation of c-Abl and p53 is associated with α-synucleinopathy. Figure S3. Activation of Mdm2 in A53TαSyn Tg Mice brainstem (BST). Figure S4. Increased cytosolic p53 in TgA53T neurons. Figure S5. Colocalization of p62 accumulation with pS129αS in TgA53T. Figure S6. c-Abl activity and autophagy in HEK-293 cells. Figure S7. c-Abl activity modulates nutrient starvation induced autophagy. Figure S8. Constitutively active c-Abl suppresses Mdm2 levels and increases p53 levels. Figure S9. Increased mTOR activation with c-Abl activation. Figure S10. c-Abl-dependent effects on mTOR and AMPK is blocked by inhibition of p53. Figure S11. Inhibition of c-Abl by nilotinib (Nilo) modulate basal autophagy, in neuronal cells. Figure S12. c-Abl-dependent effects on mTOR and AMPK. Figure S13. Nilotinib treatment decreases activation of astrocyte and microglia in the TgA53T Cerebellum and Brainstem. Figure S14. Nilotinib treatment is not able to inhibit c-Abl in the TgA53T spinal cord. Figure S15. Nilotinib treatment is not able to change αS pathology or attenuates autophagy deficit in spinal cord. Figure S16. Nilotinib treatment is not able to inhibit astrocyte/microglia activation in spinal cord. Table S1. Antibodies used for Western blotting and immunohistochemistry. [file 13024_2020_364_MOESM1_ESM.zip › 13024_2020_364_MOESM1_ESM/Figure S15.TIF]

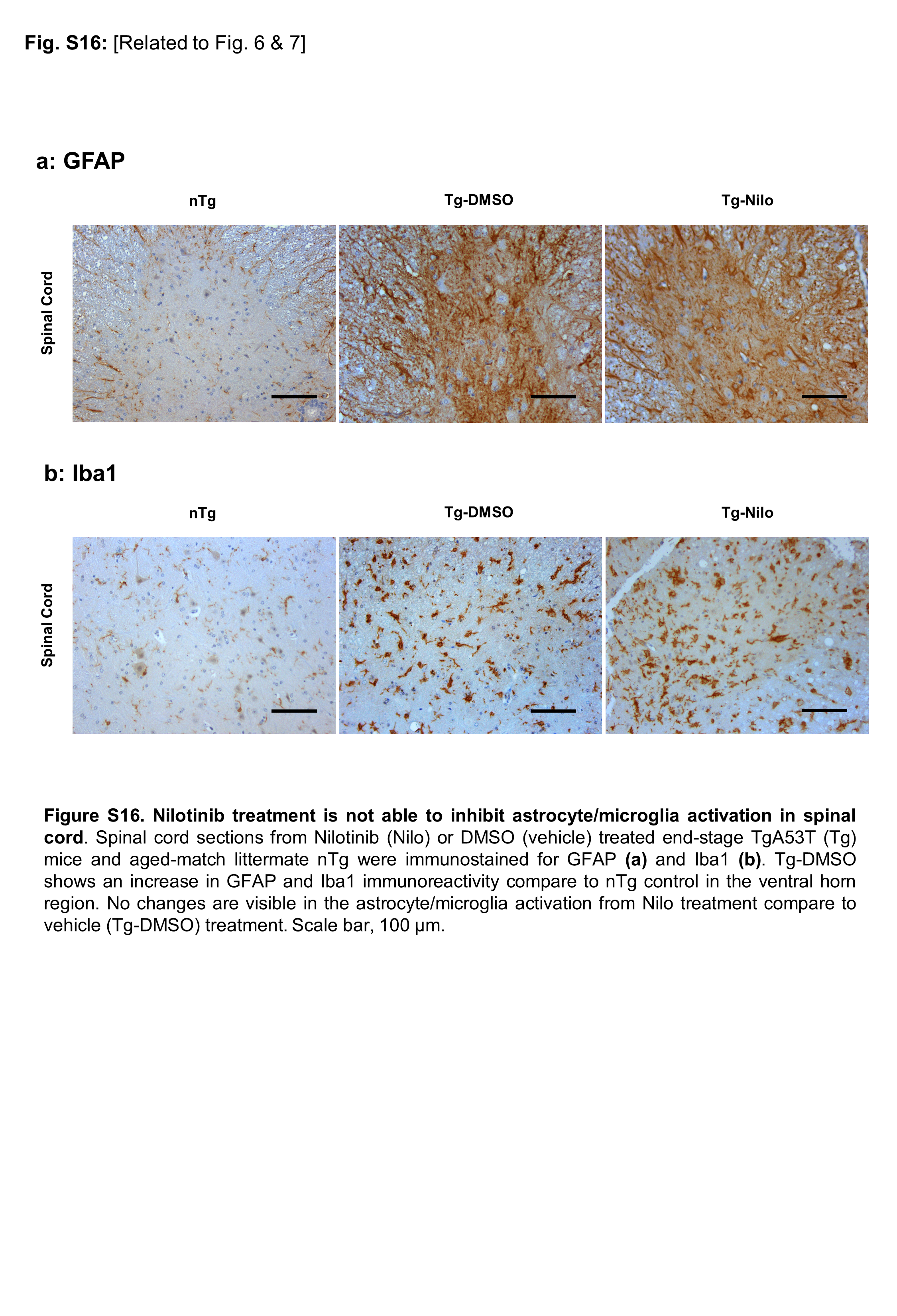

Supplement: Supplementary file 1 — Additional file 1: Figure S1. Increased 3-nitrotyrosine (3-NT) immunoreactivity in TgA53T neurons indicate neuronal oxidative stress. Figure S2. Activation of c-Abl and p53 is associated with α-synucleinopathy. Figure S3. Activation of Mdm2 in A53TαSyn Tg Mice brainstem (BST). Figure S4. Increased cytosolic p53 in TgA53T neurons. Figure S5. Colocalization of p62 accumulation with pS129αS in TgA53T. Figure S6. c-Abl activity and autophagy in HEK-293 cells. Figure S7. c-Abl activity modulates nutrient starvation induced autophagy. Figure S8. Constitutively active c-Abl suppresses Mdm2 levels and increases p53 levels. Figure S9. Increased mTOR activation with c-Abl activation. Figure S10. c-Abl-dependent effects on mTOR and AMPK is blocked by inhibition of p53. Figure S11. Inhibition of c-Abl by nilotinib (Nilo) modulate basal autophagy, in neuronal cells. Figure S12. c-Abl-dependent effects on mTOR and AMPK. Figure S13. Nilotinib treatment decreases activation of astrocyte and microglia in the TgA53T Cerebellum and Brainstem. Figure S14. Nilotinib treatment is not able to inhibit c-Abl in the TgA53T spinal cord. Figure S15. Nilotinib treatment is not able to change αS pathology or attenuates autophagy deficit in spinal cord. Figure S16. Nilotinib treatment is not able to inhibit astrocyte/microglia activation in spinal cord. Table S1. Antibodies used for Western blotting and immunohistochemistry. [file 13024_2020_364_MOESM1_ESM.zip › 13024_2020_364_MOESM1_ESM/Figure S16.TIF]

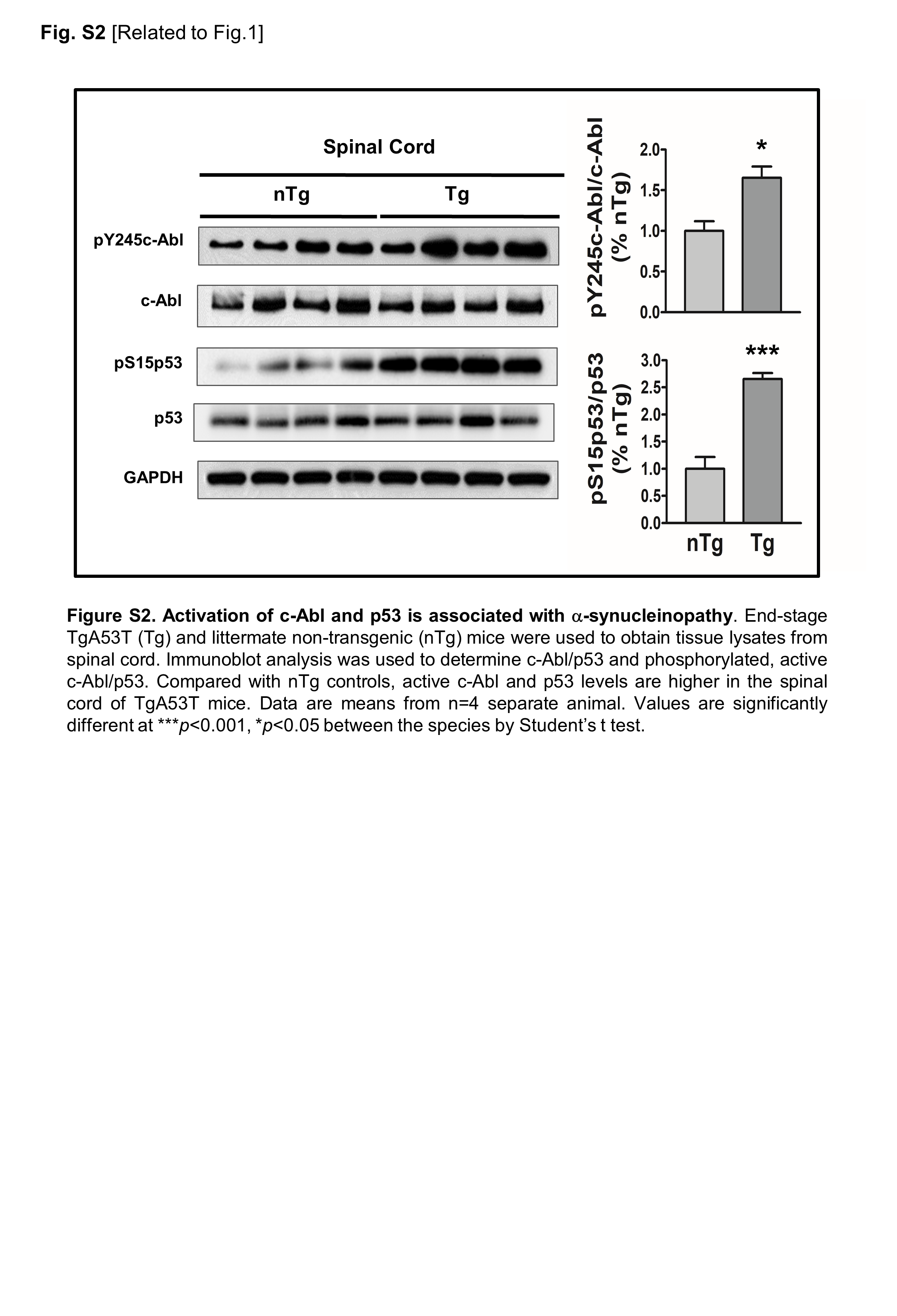

Supplement: Supplementary file 1 — Additional file 1: Figure S1. Increased 3-nitrotyrosine (3-NT) immunoreactivity in TgA53T neurons indicate neuronal oxidative stress. Figure S2. Activation of c-Abl and p53 is associated with α-synucleinopathy. Figure S3. Activation of Mdm2 in A53TαSyn Tg Mice brainstem (BST). Figure S4. Increased cytosolic p53 in TgA53T neurons. Figure S5. Colocalization of p62 accumulation with pS129αS in TgA53T. Figure S6. c-Abl activity and autophagy in HEK-293 cells. Figure S7. c-Abl activity modulates nutrient starvation induced autophagy. Figure S8. Constitutively active c-Abl suppresses Mdm2 levels and increases p53 levels. Figure S9. Increased mTOR activation with c-Abl activation. Figure S10. c-Abl-dependent effects on mTOR and AMPK is blocked by inhibition of p53. Figure S11. Inhibition of c-Abl by nilotinib (Nilo) modulate basal autophagy, in neuronal cells. Figure S12. c-Abl-dependent effects on mTOR and AMPK. Figure S13. Nilotinib treatment decreases activation of astrocyte and microglia in the TgA53T Cerebellum and Brainstem. Figure S14. Nilotinib treatment is not able to inhibit c-Abl in the TgA53T spinal cord. Figure S15. Nilotinib treatment is not able to change αS pathology or attenuates autophagy deficit in spinal cord. Figure S16. Nilotinib treatment is not able to inhibit astrocyte/microglia activation in spinal cord. Table S1. Antibodies used for Western blotting and immunohistochemistry. [file 13024_2020_364_MOESM1_ESM.zip › 13024_2020_364_MOESM1_ESM/Figure S2.TIF]

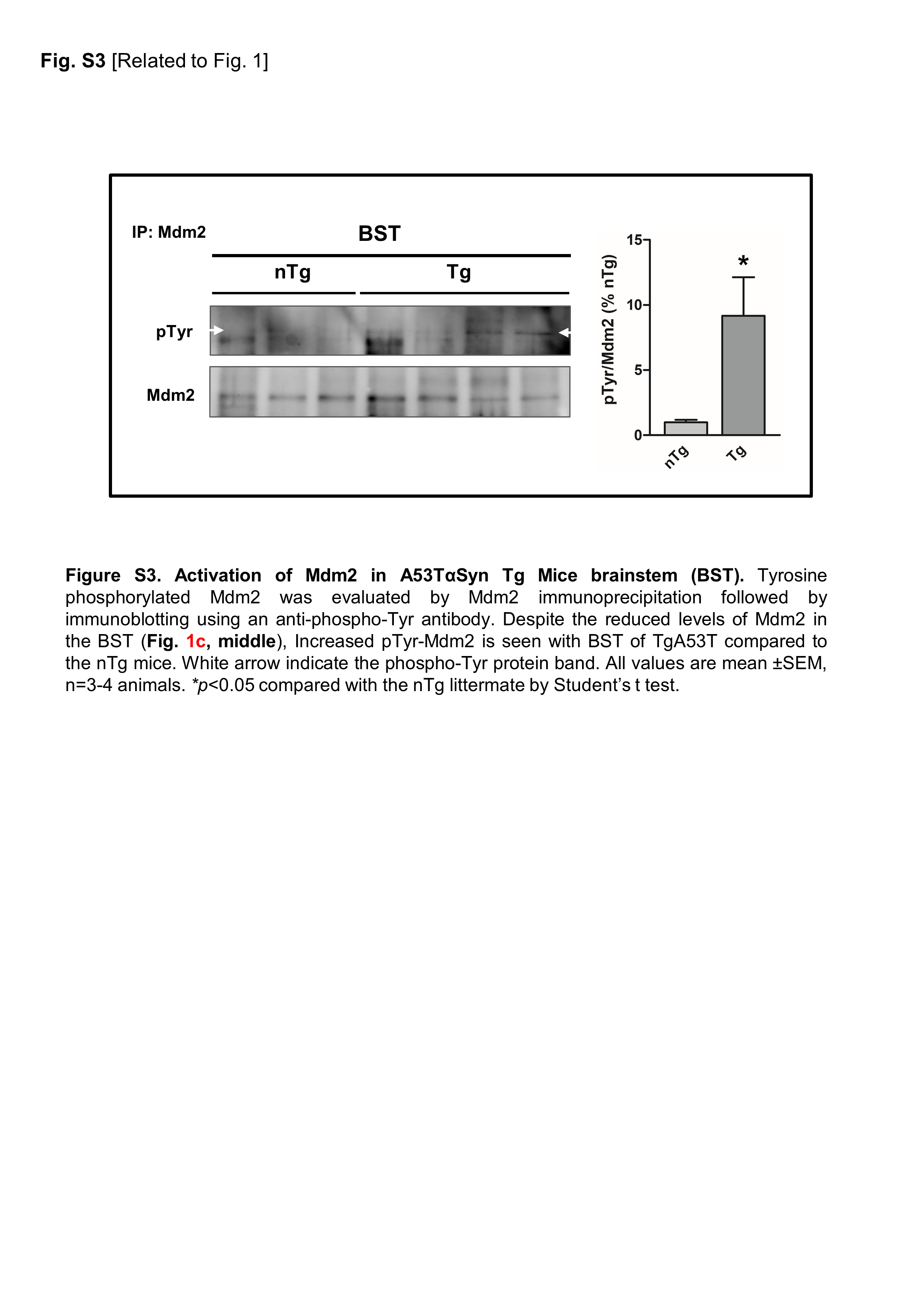

Supplement: Supplementary file 1 — Additional file 1: Figure S1. Increased 3-nitrotyrosine (3-NT) immunoreactivity in TgA53T neurons indicate neuronal oxidative stress. Figure S2. Activation of c-Abl and p53 is associated with α-synucleinopathy. Figure S3. Activation of Mdm2 in A53TαSyn Tg Mice brainstem (BST). Figure S4. Increased cytosolic p53 in TgA53T neurons. Figure S5. Colocalization of p62 accumulation with pS129αS in TgA53T. Figure S6. c-Abl activity and autophagy in HEK-293 cells. Figure S7. c-Abl activity modulates nutrient starvation induced autophagy. Figure S8. Constitutively active c-Abl suppresses Mdm2 levels and increases p53 levels. Figure S9. Increased mTOR activation with c-Abl activation. Figure S10. c-Abl-dependent effects on mTOR and AMPK is blocked by inhibition of p53. Figure S11. Inhibition of c-Abl by nilotinib (Nilo) modulate basal autophagy, in neuronal cells. Figure S12. c-Abl-dependent effects on mTOR and AMPK. Figure S13. Nilotinib treatment decreases activation of astrocyte and microglia in the TgA53T Cerebellum and Brainstem. Figure S14. Nilotinib treatment is not able to inhibit c-Abl in the TgA53T spinal cord. Figure S15. Nilotinib treatment is not able to change αS pathology or attenuates autophagy deficit in spinal cord. Figure S16. Nilotinib treatment is not able to inhibit astrocyte/microglia activation in spinal cord. Table S1. Antibodies used for Western blotting and immunohistochemistry. [file 13024_2020_364_MOESM1_ESM.zip › 13024_2020_364_MOESM1_ESM/Figure S3.TIF]

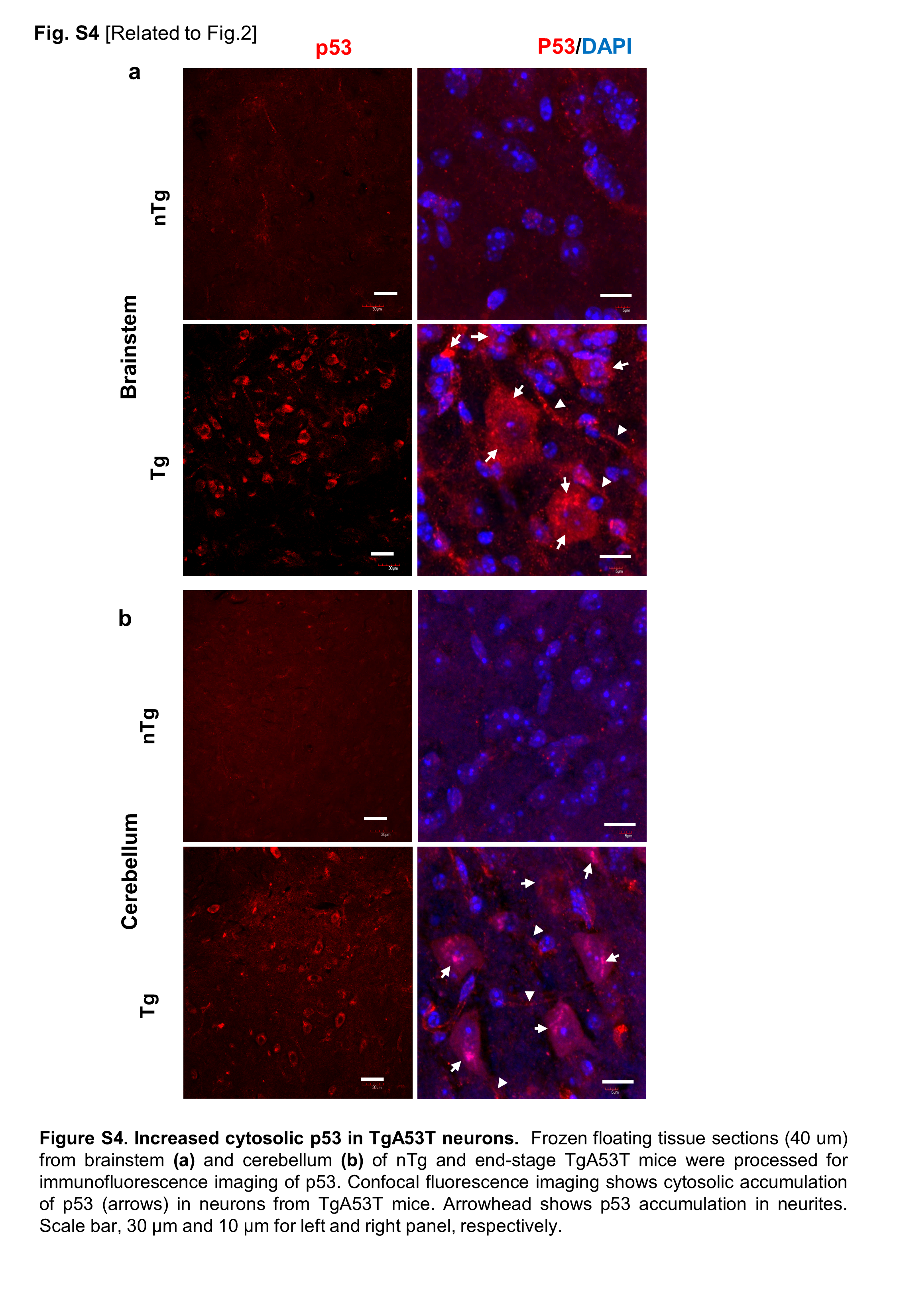

Supplement: Supplementary file 1 — Additional file 1: Figure S1. Increased 3-nitrotyrosine (3-NT) immunoreactivity in TgA53T neurons indicate neuronal oxidative stress. Figure S2. Activation of c-Abl and p53 is associated with α-synucleinopathy. Figure S3. Activation of Mdm2 in A53TαSyn Tg Mice brainstem (BST). Figure S4. Increased cytosolic p53 in TgA53T neurons. Figure S5. Colocalization of p62 accumulation with pS129αS in TgA53T. Figure S6. c-Abl activity and autophagy in HEK-293 cells. Figure S7. c-Abl activity modulates nutrient starvation induced autophagy. Figure S8. Constitutively active c-Abl suppresses Mdm2 levels and increases p53 levels. Figure S9. Increased mTOR activation with c-Abl activation. Figure S10. c-Abl-dependent effects on mTOR and AMPK is blocked by inhibition of p53. Figure S11. Inhibition of c-Abl by nilotinib (Nilo) modulate basal autophagy, in neuronal cells. Figure S12. c-Abl-dependent effects on mTOR and AMPK. Figure S13. Nilotinib treatment decreases activation of astrocyte and microglia in the TgA53T Cerebellum and Brainstem. Figure S14. Nilotinib treatment is not able to inhibit c-Abl in the TgA53T spinal cord. Figure S15. Nilotinib treatment is not able to change αS pathology or attenuates autophagy deficit in spinal cord. Figure S16. Nilotinib treatment is not able to inhibit astrocyte/microglia activation in spinal cord. Table S1. Antibodies used for Western blotting and immunohistochemistry. [file 13024_2020_364_MOESM1_ESM.zip › 13024_2020_364_MOESM1_ESM/Figure S4.TIF]

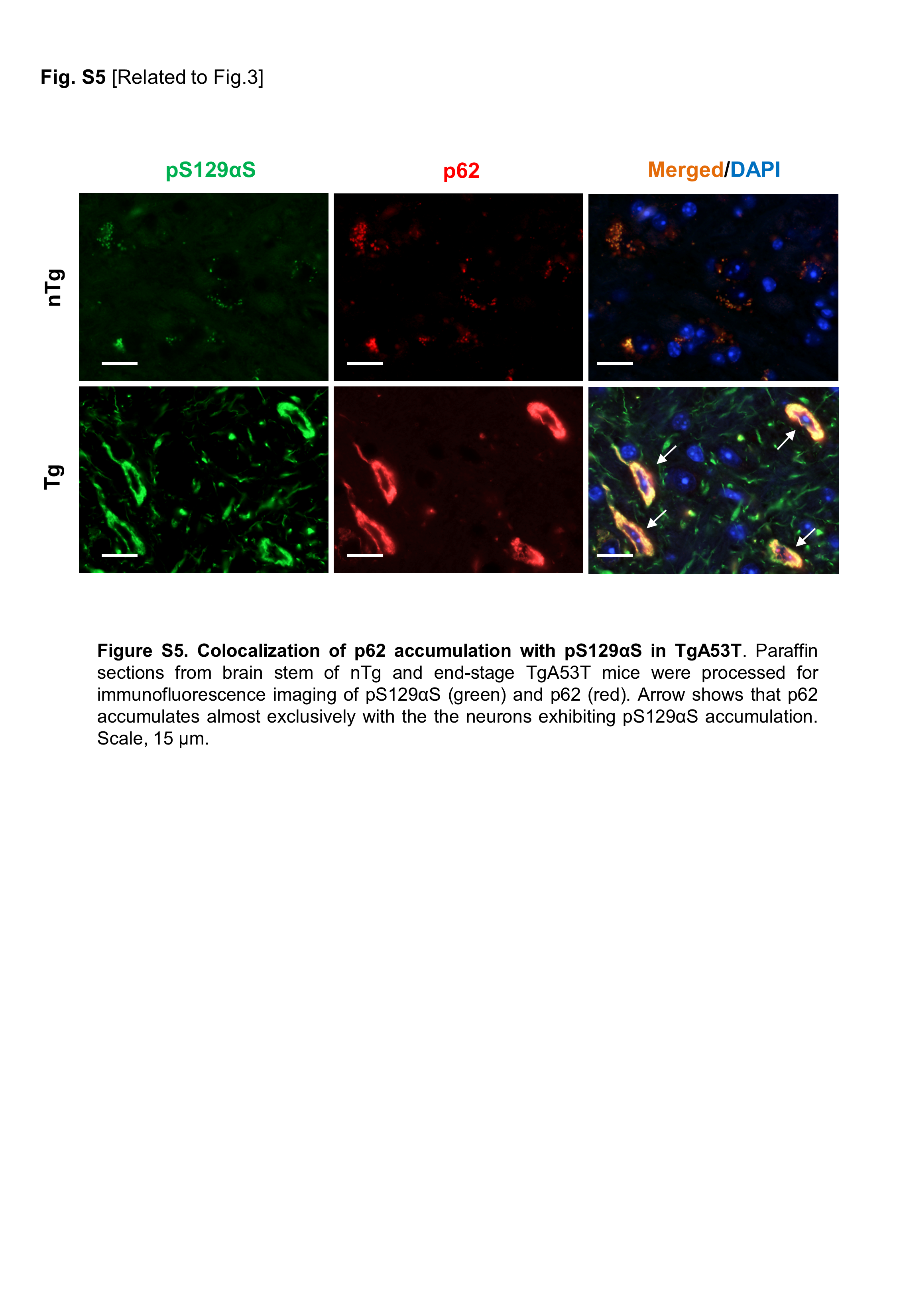

Supplement: Supplementary file 1 — Additional file 1: Figure S1. Increased 3-nitrotyrosine (3-NT) immunoreactivity in TgA53T neurons indicate neuronal oxidative stress. Figure S2. Activation of c-Abl and p53 is associated with α-synucleinopathy. Figure S3. Activation of Mdm2 in A53TαSyn Tg Mice brainstem (BST). Figure S4. Increased cytosolic p53 in TgA53T neurons. Figure S5. Colocalization of p62 accumulation with pS129αS in TgA53T. Figure S6. c-Abl activity and autophagy in HEK-293 cells. Figure S7. c-Abl activity modulates nutrient starvation induced autophagy. Figure S8. Constitutively active c-Abl suppresses Mdm2 levels and increases p53 levels. Figure S9. Increased mTOR activation with c-Abl activation. Figure S10. c-Abl-dependent effects on mTOR and AMPK is blocked by inhibition of p53. Figure S11. Inhibition of c-Abl by nilotinib (Nilo) modulate basal autophagy, in neuronal cells. Figure S12. c-Abl-dependent effects on mTOR and AMPK. Figure S13. Nilotinib treatment decreases activation of astrocyte and microglia in the TgA53T Cerebellum and Brainstem. Figure S14. Nilotinib treatment is not able to inhibit c-Abl in the TgA53T spinal cord. Figure S15. Nilotinib treatment is not able to change αS pathology or attenuates autophagy deficit in spinal cord. Figure S16. Nilotinib treatment is not able to inhibit astrocyte/microglia activation in spinal cord. Table S1. Antibodies used for Western blotting and immunohistochemistry. [file 13024_2020_364_MOESM1_ESM.zip › 13024_2020_364_MOESM1_ESM/Figure S5.TIF]

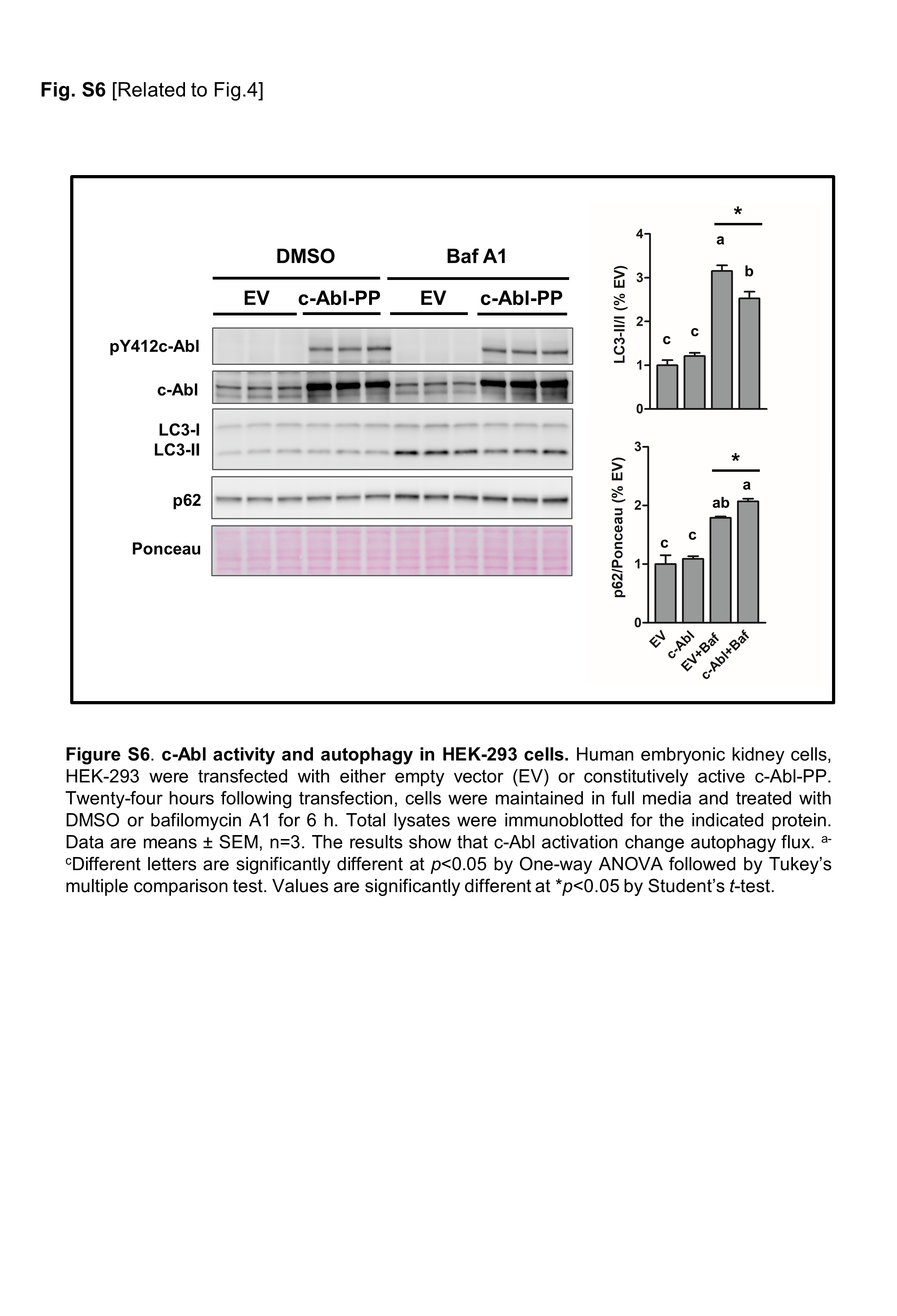

Supplement: Supplementary file 1 — Additional file 1: Figure S1. Increased 3-nitrotyrosine (3-NT) immunoreactivity in TgA53T neurons indicate neuronal oxidative stress. Figure S2. Activation of c-Abl and p53 is associated with α-synucleinopathy. Figure S3. Activation of Mdm2 in A53TαSyn Tg Mice brainstem (BST). Figure S4. Increased cytosolic p53 in TgA53T neurons. Figure S5. Colocalization of p62 accumulation with pS129αS in TgA53T. Figure S6. c-Abl activity and autophagy in HEK-293 cells. Figure S7. c-Abl activity modulates nutrient starvation induced autophagy. Figure S8. Constitutively active c-Abl suppresses Mdm2 levels and increases p53 levels. Figure S9. Increased mTOR activation with c-Abl activation. Figure S10. c-Abl-dependent effects on mTOR and AMPK is blocked by inhibition of p53. Figure S11. Inhibition of c-Abl by nilotinib (Nilo) modulate basal autophagy, in neuronal cells. Figure S12. c-Abl-dependent effects on mTOR and AMPK. Figure S13. Nilotinib treatment decreases activation of astrocyte and microglia in the TgA53T Cerebellum and Brainstem. Figure S14. Nilotinib treatment is not able to inhibit c-Abl in the TgA53T spinal cord. Figure S15. Nilotinib treatment is not able to change αS pathology or attenuates autophagy deficit in spinal cord. Figure S16. Nilotinib treatment is not able to inhibit astrocyte/microglia activation in spinal cord. Table S1. Antibodies used for Western blotting and immunohistochemistry. [file 13024_2020_364_MOESM1_ESM.zip › 13024_2020_364_MOESM1_ESM/Figure S6.TIF]

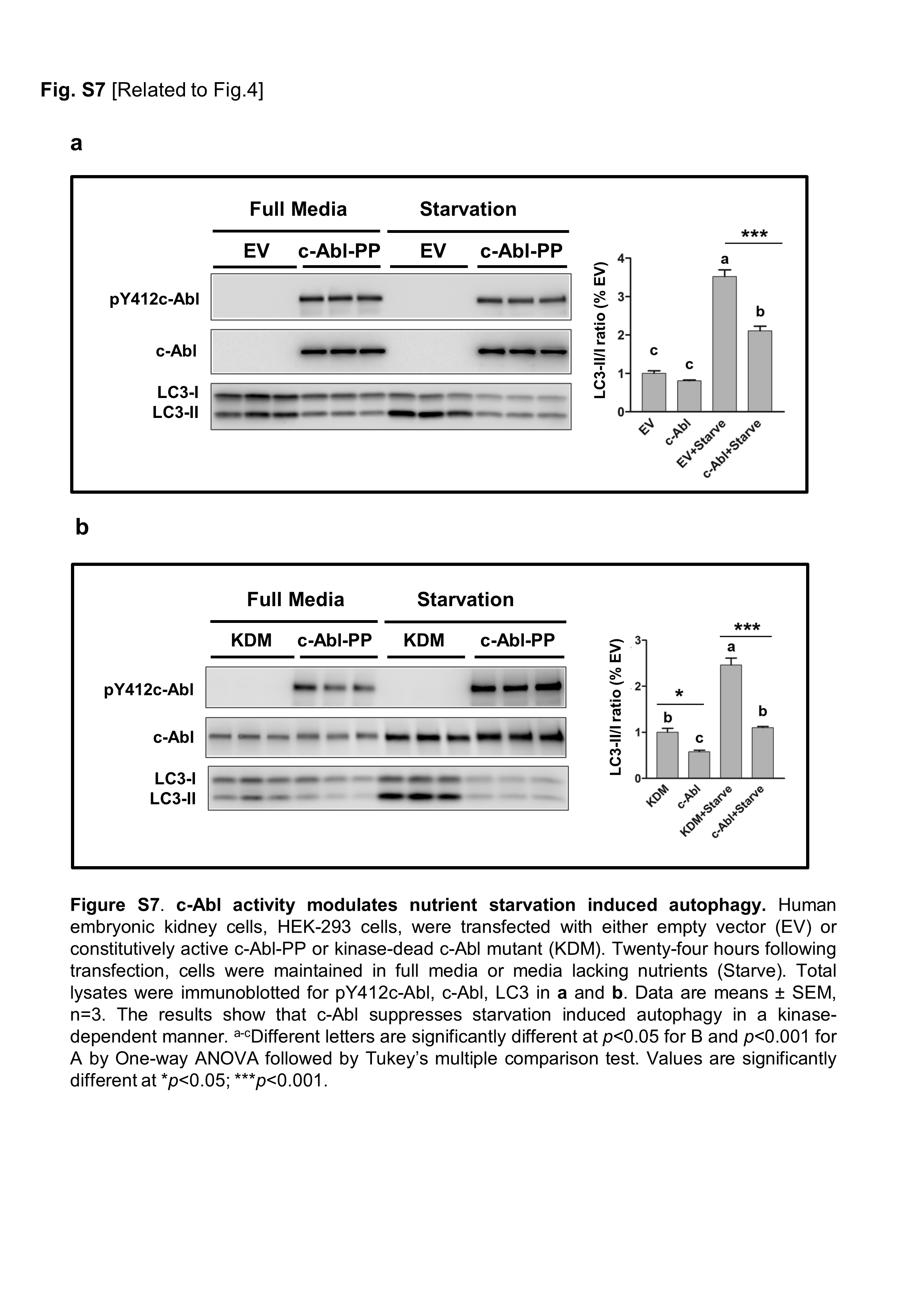

Supplement: Supplementary file 1 — Additional file 1: Figure S1. Increased 3-nitrotyrosine (3-NT) immunoreactivity in TgA53T neurons indicate neuronal oxidative stress. Figure S2. Activation of c-Abl and p53 is associated with α-synucleinopathy. Figure S3. Activation of Mdm2 in A53TαSyn Tg Mice brainstem (BST). Figure S4. Increased cytosolic p53 in TgA53T neurons. Figure S5. Colocalization of p62 accumulation with pS129αS in TgA53T. Figure S6. c-Abl activity and autophagy in HEK-293 cells. Figure S7. c-Abl activity modulates nutrient starvation induced autophagy. Figure S8. Constitutively active c-Abl suppresses Mdm2 levels and increases p53 levels. Figure S9. Increased mTOR activation with c-Abl activation. Figure S10. c-Abl-dependent effects on mTOR and AMPK is blocked by inhibition of p53. Figure S11. Inhibition of c-Abl by nilotinib (Nilo) modulate basal autophagy, in neuronal cells. Figure S12. c-Abl-dependent effects on mTOR and AMPK. Figure S13. Nilotinib treatment decreases activation of astrocyte and microglia in the TgA53T Cerebellum and Brainstem. Figure S14. Nilotinib treatment is not able to inhibit c-Abl in the TgA53T spinal cord. Figure S15. Nilotinib treatment is not able to change αS pathology or attenuates autophagy deficit in spinal cord. Figure S16. Nilotinib treatment is not able to inhibit astrocyte/microglia activation in spinal cord. Table S1. Antibodies used for Western blotting and immunohistochemistry. [file 13024_2020_364_MOESM1_ESM.zip › 13024_2020_364_MOESM1_ESM/Figure S7.TIF]

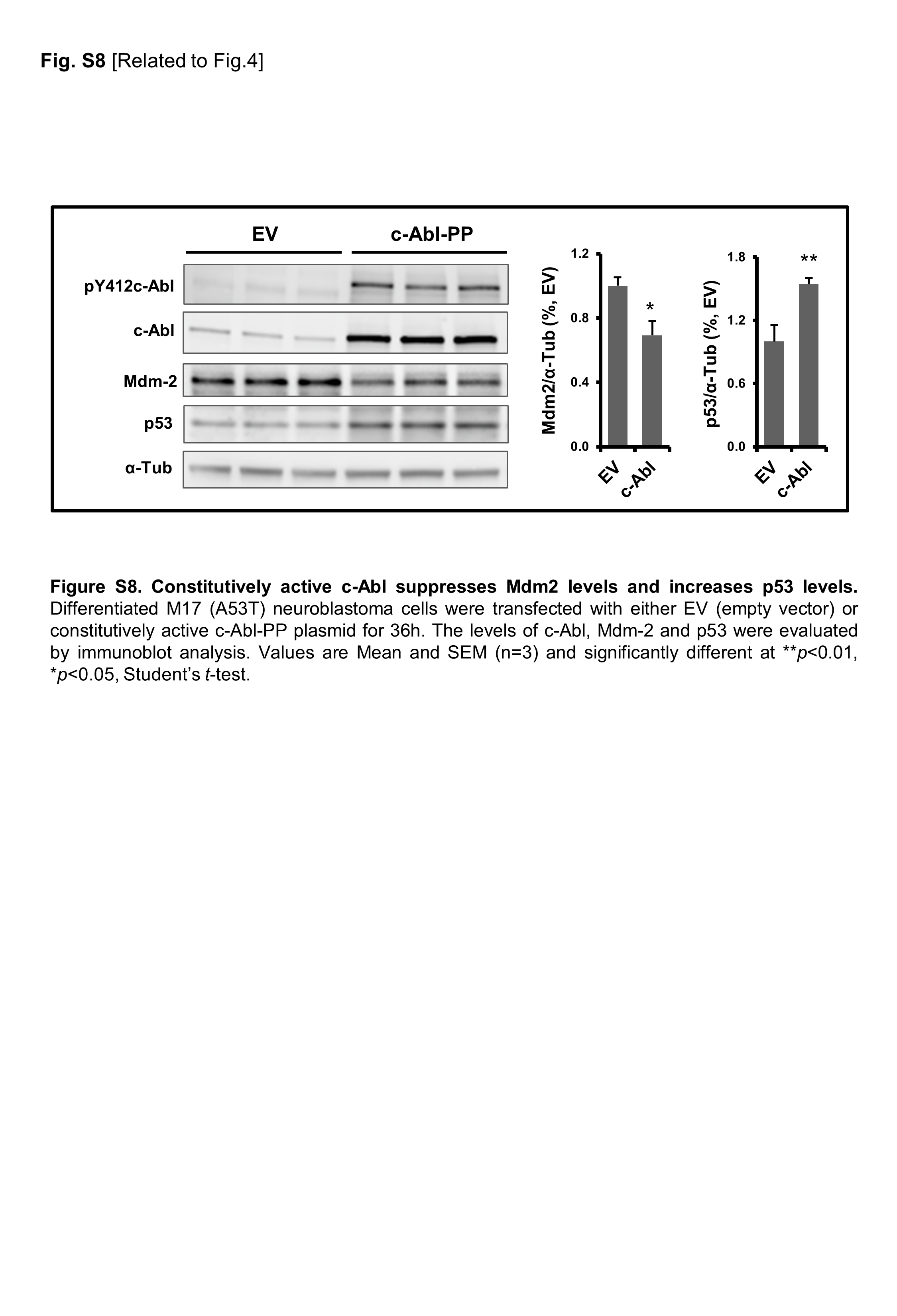

Supplement: Supplementary file 1 — Additional file 1: Figure S1. Increased 3-nitrotyrosine (3-NT) immunoreactivity in TgA53T neurons indicate neuronal oxidative stress. Figure S2. Activation of c-Abl and p53 is associated with α-synucleinopathy. Figure S3. Activation of Mdm2 in A53TαSyn Tg Mice brainstem (BST). Figure S4. Increased cytosolic p53 in TgA53T neurons. Figure S5. Colocalization of p62 accumulation with pS129αS in TgA53T. Figure S6. c-Abl activity and autophagy in HEK-293 cells. Figure S7. c-Abl activity modulates nutrient starvation induced autophagy. Figure S8. Constitutively active c-Abl suppresses Mdm2 levels and increases p53 levels. Figure S9. Increased mTOR activation with c-Abl activation. Figure S10. c-Abl-dependent effects on mTOR and AMPK is blocked by inhibition of p53. Figure S11. Inhibition of c-Abl by nilotinib (Nilo) modulate basal autophagy, in neuronal cells. Figure S12. c-Abl-dependent effects on mTOR and AMPK. Figure S13. Nilotinib treatment decreases activation of astrocyte and microglia in the TgA53T Cerebellum and Brainstem. Figure S14. Nilotinib treatment is not able to inhibit c-Abl in the TgA53T spinal cord. Figure S15. Nilotinib treatment is not able to change αS pathology or attenuates autophagy deficit in spinal cord. Figure S16. Nilotinib treatment is not able to inhibit astrocyte/microglia activation in spinal cord. Table S1. Antibodies used for Western blotting and immunohistochemistry. [file 13024_2020_364_MOESM1_ESM.zip › 13024_2020_364_MOESM1_ESM/Figure S8.TIF]

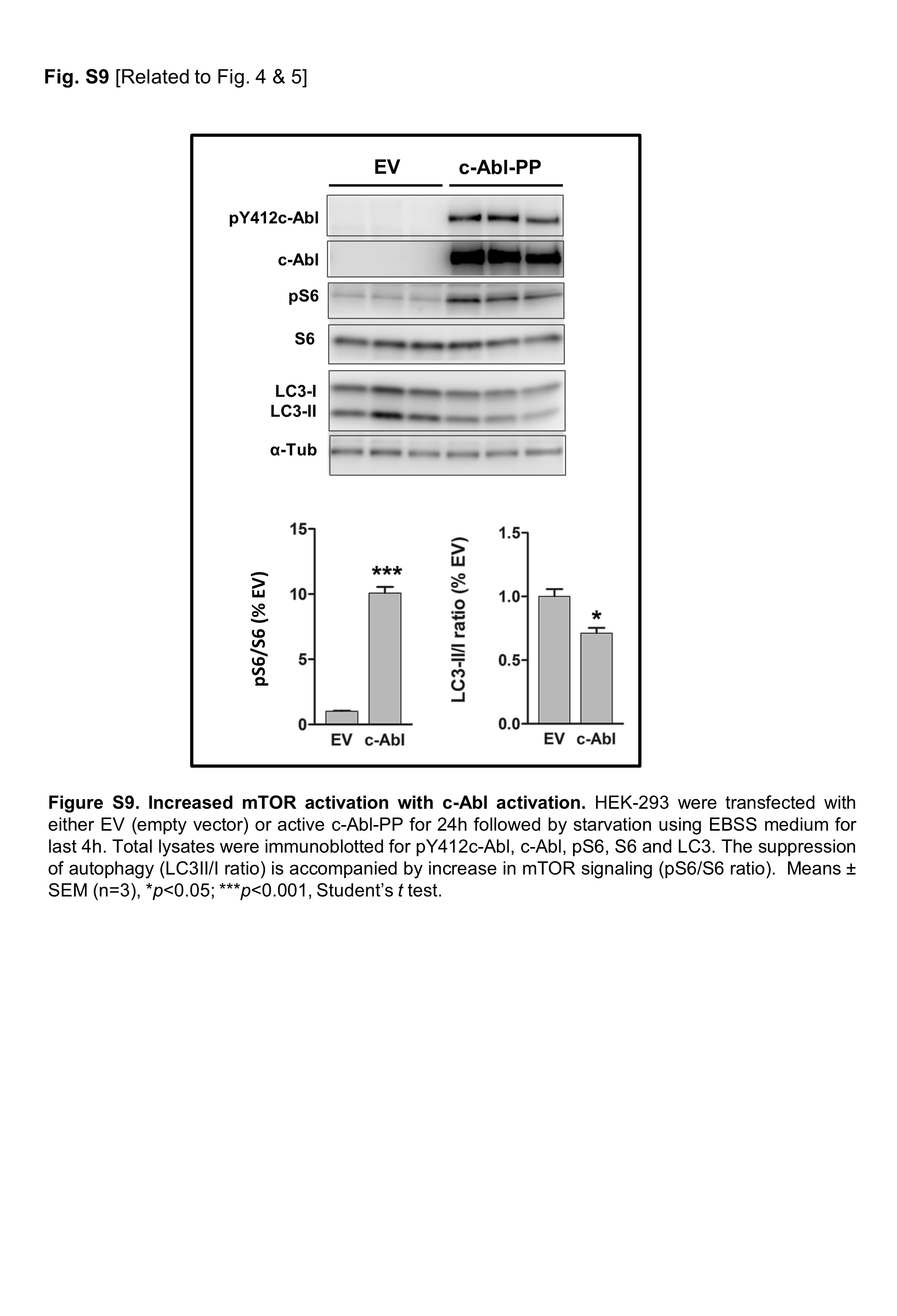

Supplement: Supplementary file 1 — Additional file 1: Figure S1. Increased 3-nitrotyrosine (3-NT) immunoreactivity in TgA53T neurons indicate neuronal oxidative stress. Figure S2. Activation of c-Abl and p53 is associated with α-synucleinopathy. Figure S3. Activation of Mdm2 in A53TαSyn Tg Mice brainstem (BST). Figure S4. Increased cytosolic p53 in TgA53T neurons. Figure S5. Colocalization of p62 accumulation with pS129αS in TgA53T. Figure S6. c-Abl activity and autophagy in HEK-293 cells. Figure S7. c-Abl activity modulates nutrient starvation induced autophagy. Figure S8. Constitutively active c-Abl suppresses Mdm2 levels and increases p53 levels. Figure S9. Increased mTOR activation with c-Abl activation. Figure S10. c-Abl-dependent effects on mTOR and AMPK is blocked by inhibition of p53. Figure S11. Inhibition of c-Abl by nilotinib (Nilo) modulate basal autophagy, in neuronal cells. Figure S12. c-Abl-dependent effects on mTOR and AMPK. Figure S13. Nilotinib treatment decreases activation of astrocyte and microglia in the TgA53T Cerebellum and Brainstem. Figure S14. Nilotinib treatment is not able to inhibit c-Abl in the TgA53T spinal cord. Figure S15. Nilotinib treatment is not able to change αS pathology or attenuates autophagy deficit in spinal cord. Figure S16. Nilotinib treatment is not able to inhibit astrocyte/microglia activation in spinal cord. Table S1. Antibodies used for Western blotting and immunohistochemistry. [file 13024_2020_364_MOESM1_ESM.zip › 13024_2020_364_MOESM1_ESM/Figure S9.TIF]

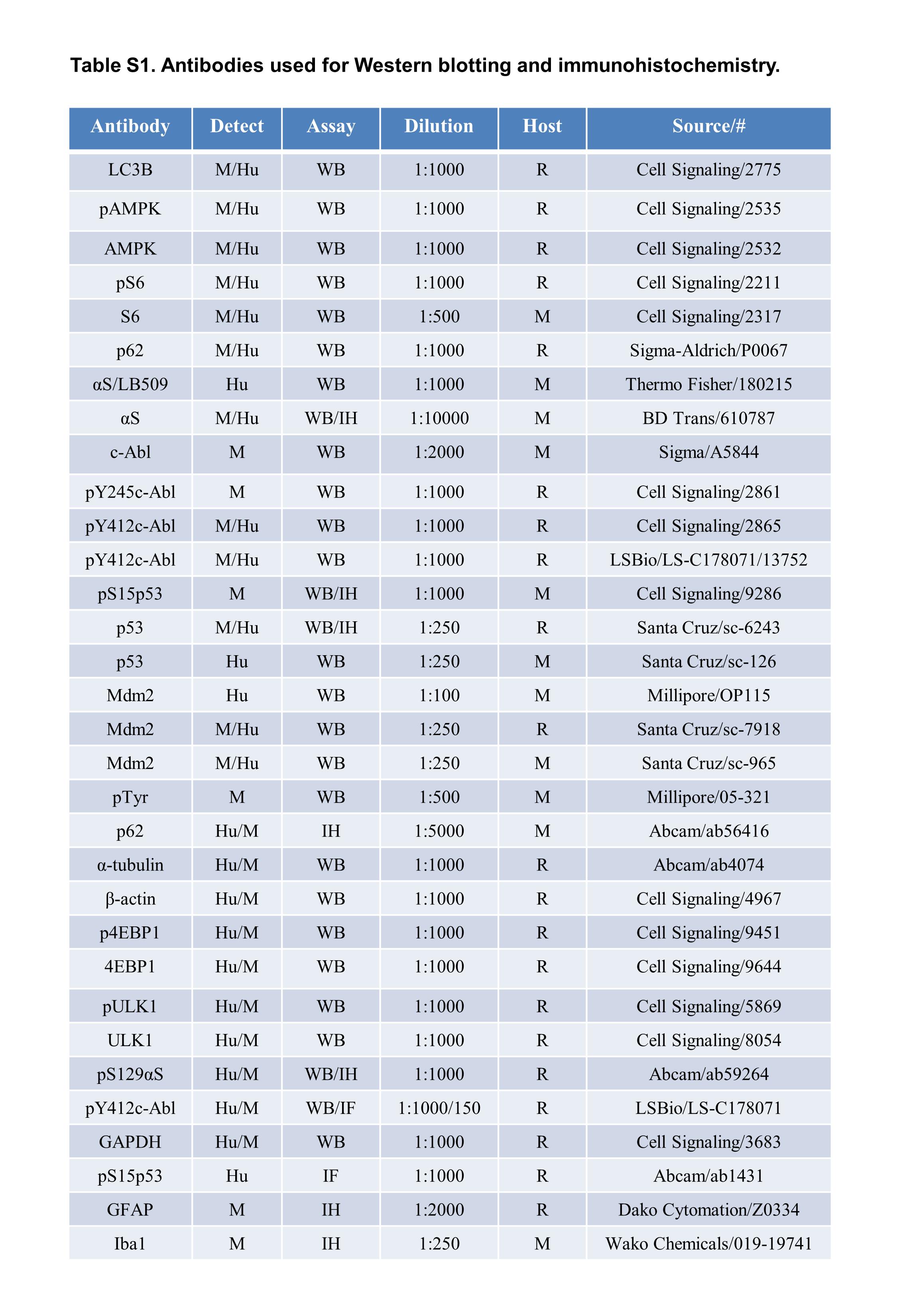

Supplement: Supplementary file 1 — Additional file 1: Figure S1. Increased 3-nitrotyrosine (3-NT) immunoreactivity in TgA53T neurons indicate neuronal oxidative stress. Figure S2. Activation of c-Abl and p53 is associated with α-synucleinopathy. Figure S3. Activation of Mdm2 in A53TαSyn Tg Mice brainstem (BST). Figure S4. Increased cytosolic p53 in TgA53T neurons. Figure S5. Colocalization of p62 accumulation with pS129αS in TgA53T. Figure S6. c-Abl activity and autophagy in HEK-293 cells. Figure S7. c-Abl activity modulates nutrient starvation induced autophagy. Figure S8. Constitutively active c-Abl suppresses Mdm2 levels and increases p53 levels. Figure S9. Increased mTOR activation with c-Abl activation. Figure S10. c-Abl-dependent effects on mTOR and AMPK is blocked by inhibition of p53. Figure S11. Inhibition of c-Abl by nilotinib (Nilo) modulate basal autophagy, in neuronal cells. Figure S12. c-Abl-dependent effects on mTOR and AMPK. Figure S13. Nilotinib treatment decreases activation of astrocyte and microglia in the TgA53T Cerebellum and Brainstem. Figure S14. Nilotinib treatment is not able to inhibit c-Abl in the TgA53T spinal cord. Figure S15. Nilotinib treatment is not able to change αS pathology or attenuates autophagy deficit in spinal cord. Figure S16. Nilotinib treatment is not able to inhibit astrocyte/microglia activation in spinal cord. Table S1. Antibodies used for Western blotting and immunohistochemistry. [file 13024_2020_364_MOESM1_ESM.zip › 13024_2020_364_MOESM1_ESM/Table S1.TIF]
